# Ribosome biogenesis during cell cycle arrest fuels EMT in development and disease

**DOI:** 10.1038/s41467-019-10100-8

**Published:** 2019-05-08

**Authors:** Varsha Prakash, Brittany B. Carson, Jennifer M. Feenstra, Randall A. Dass, Petra Sekyrova, Ayuko Hoshino, Julian Petersen, Yuan Guo, Matthew M. Parks, Chad M. Kurylo, Jake E. Batchelder, Kristian Haller, Ayako Hashimoto, Helene Rundqivst, John S. Condeelis, C. David Allis, Denis Drygin, M. Angela Nieto, Michael Andäng, Piergiorgio Percipalle, Jonas Bergh, Igor Adameyko, Ann-Kristin Östlund Farrants, Johan Hartman, David Lyden, Kristian Pietras, Scott C. Blanchard, C. Theresa Vincent

**Affiliations:** 1 Department of Physiology and Pharmacology, Karolinska Institutet, 171 77 Stockholm, Sweden; 2 Department of Immunology, Genetics and Pathology, Uppsala University, 751 85 Uppsala, Sweden; 3 Department of Physiology and Biophysics, Weill Cornell Medicine, New York, NY 10065 USA; 4 Meyer Cancer Center, Weill Cornell Medicine, New York, NY 10065 USA; 5 Department of Pediatrics and Cell and Developmental Biology, Weill Cornell Medicine College, New York, NY 10065 USA; 6 Department for Brain Research, Medical University of Vienna, 1090 Vienna, Austria; 7 Department of Molecular Biosciences, The Wenner-Gren Institute, Stockholm University, S-10691 Stockholm, Sweden; 8 Department of Laboratory Medicine, Center for Molecular Pathology, Lund University, Lund, SE-223 81 Sweden; 9 Department of Cell and Molecular Biology, Karolinska Institute, Stockholm, SE-171 77 Sweden; 10 Gruss Lipper Biophotonics Center, Albert Einstein College of Medicine, Bronx, 10461 NY USA; 11 Department of Pathology, Montefiore Medical Center, Bronx, 10461 NY USA; 12 Laboratory of Chromatin Biology and Epigenetics, The Rockefeller University, New York, NY 10065 USA; 13 Pimera, Inc, 3210 Merryfield Row, San Diego, CA 92121 USA; 14 Instituto de Neurociencias, CSIC-UMH, Alicante, 03550 Spain; 15 Science Division, Biology Program, New York University Abu Dhabi, Abu Dhabi, 129188 UAE; 16 Department of Oncology and Pathology, Karolinska Institutet and University Hospital, S-171 76 Solna, Sweden; 17 Tri-Institutional Training Program in Chemical Biology, Weill Cornell Medicine, New York, NY 10065 USA

**Keywords:** Breast cancer, Cell migration

## Abstract

Ribosome biogenesis is a canonical hallmark of cell growth and proliferation. Here we show that execution of Epithelial-to-Mesenchymal Transition (EMT), a migratory cellular program associated with development and tumor metastasis, is fueled by upregulation of ribosome biogenesis during G1/S arrest. This unexpected EMT feature is independent of species and initiating signal, and is accompanied by release of the repressive nucleolar chromatin remodeling complex (NoRC) from rDNA, together with recruitment of the EMT-driving transcription factor Snai1 (Snail1), RNA Polymerase I (Pol I) and the Upstream Binding Factor (UBF). EMT-associated ribosome biogenesis is also coincident with increased nucleolar recruitment of Rictor, an essential component of the EMT-promoting mammalian target of rapamycin complex 2 (mTORC2). Inhibition of rRNA synthesis in vivo differentiates primary tumors to a benign, Estrogen Receptor-alpha (ERα) positive, Rictor-negative phenotype and reduces metastasis. These findings implicate the EMT-associated ribosome biogenesis program with cellular plasticity, de-differentiation, cancer progression and metastatic disease.

## Abstract (web summary)

Ribosomal DNA transcription is essential for cell growth and division. Here, the authors show that EMT is accompanied by Snail binding to rDNA operons and Rictor association with nucleoli to fuel an induction of ribosome biogenesis during G1/S cell cycle arrest and inhibition of ribosome biogenesis hampers EMT, differentiates primary tumors and reduces metastasis.

## Introduction

Metastasis is the leading cause of breast cancer-associated mortality^1^. The mechanisms underlying metastasis, including the orchestrated programs coordinating the migration and dissemination of primary tumor cells to distal tissues, remain unclear^2^. The EMT program is an exemplar of cellular plasticity that de-differentiates epithelial cells to a stem-like mesenchymal phenotype to promote cell migration in development and disease^3–5^. Like other differentiation and dedifferentiation programs^6^, EMT is characterized by cell cycle arrest and the cessation of proliferation^7,8^.

Pervasive reprogramming of both transcription and translation during EMT endows the cell with pro-migratory, invasive properties^3–5,9^. Transcriptional changes are mediated, in part, by EMT-associated transcription factors, including Snail1/2, Smads, ZEB1, and Twist^3–5^. The EMT program is implicated in the initiating steps of malignancy and the resistance of tumor cell subpopulations to anti-proliferative chemotherapies^3,10^. Correspondingly, a deeper understanding of the regulation and execution of EMT has the potential to expand our knowledge of disease progression and the repertoire of treatment strategies used to combat metastatic disease.

EMT can be induced by a variety of physiological signals^3,5^. The induction of EMT by transforming growth factor beta (TGFβ) occurs via activation of Smad-dependent and Smad-independent signaling pathways^11,12^. Smad-independent signaling can activate mTORC2, a multicomponent protein complex that includes the adaptor protein Rictor and the mammalian stress-activated protein kinase interaction protein 1 (mSin1)^12^. mTORC2 activation is linked to its association with the ribosome^13,14^, the two-subunit RNA–protein complex responsible for cellular protein synthesis.

Ribosome biogenesis occurs in the nucleolus and is initiated by transcription of rDNA operons by RNA polymerase I (Pol I). The three major rRNA constituents of the ribosome (5.8S, 18S and 28S rRNAs) are generated by Pol I^15^. The fourth rRNA component (5S rRNA), as well as the transfer RNA (tRNA) substrates used in protein synthesis, are transcribed by Pol III^16^.

Active ribosome biogenesis is regulated in a cell cycle-dependent manner^15^ and is typically associated with cell growth and division^17^. Ribosome biogenesis increases the size of nucleolar organizing regions (NORs) which have long been used as a marker of tumor cell proliferation that negatively correlates with patient survival^17–19^. The induction of rDNA transcription is also associated with cellular plasticity, dedifferentiation, and stemness^20^. Given that approximately half of all rDNA operons are silenced in differentiated cells by the repressive TTF-I interacting protein 5 (TIP5)-associated, NoRC^21^, we hypothesized that the de-differentiating EMT program may be accompanied by changes in rDNA transcription. In this context, we set out to examine whether ribosome biogenesis contributes to EMT and metastatic cancer progression.

Here, we show that the induction of ribosome biogenesis is a general feature of the EMT program. Activation of ribosome biogenesis, the mesenchymal gene expression program, and a migratory phenotype is concurrent with NoRC dissociation from rDNA, together with increased expression and association of Pol I, the Pol I-transcription factor UBF, and the EMT-promoting transcription factor Snail1, with rDNA. Consistent with ribosome biogenesis being a necessary feature of EMT, pharmacological inhibition and genetic knockdown of Pol I lowered the abundance of pro-invasive mesenchymal proteins and reduced cellular invasiveness. In line with mTORC2 activation driving EMT through physical interactions with the ribosome^13,14^, we further show that TGFβ-driven EMT induces rRNA-dependent association of Rictor with nucleoli.

In mouse models of metastatic breast cancer, the suppression of Pol I-mediated transcription by CX-5461, a clinical-stage small molecule that inhibits Pol I assembly at rDNA operons^22–24^, reduced the metastatic potential of primary tumors as well as metastatic seeding. These impacts correlated with decreased Snail1 and Rictor abundance and increased Cytokeratin 8/18 (CK8/18) and Estrogen Receptor-alpha (ERα) protein levels, indicative of tumor differentiation. Elevated rRNA synthesis was also evidenced in invasive, dedifferentiated human breast cancers, including triple-negative tissues, compared to ERα+ tumor tissues and normal breast tissues. These findings collectively indicate that the EMT-associated ribosome biogenesis program fuels cellular plasticity and migration and that targeting this program can induce tumor differentiation in a manner that may potentially restore or enhance patient responsiveness to established endocrine therapies.

## Results

### rRNA synthesis is induced in vitro and in vivo during EMT

**Fig. 1:**
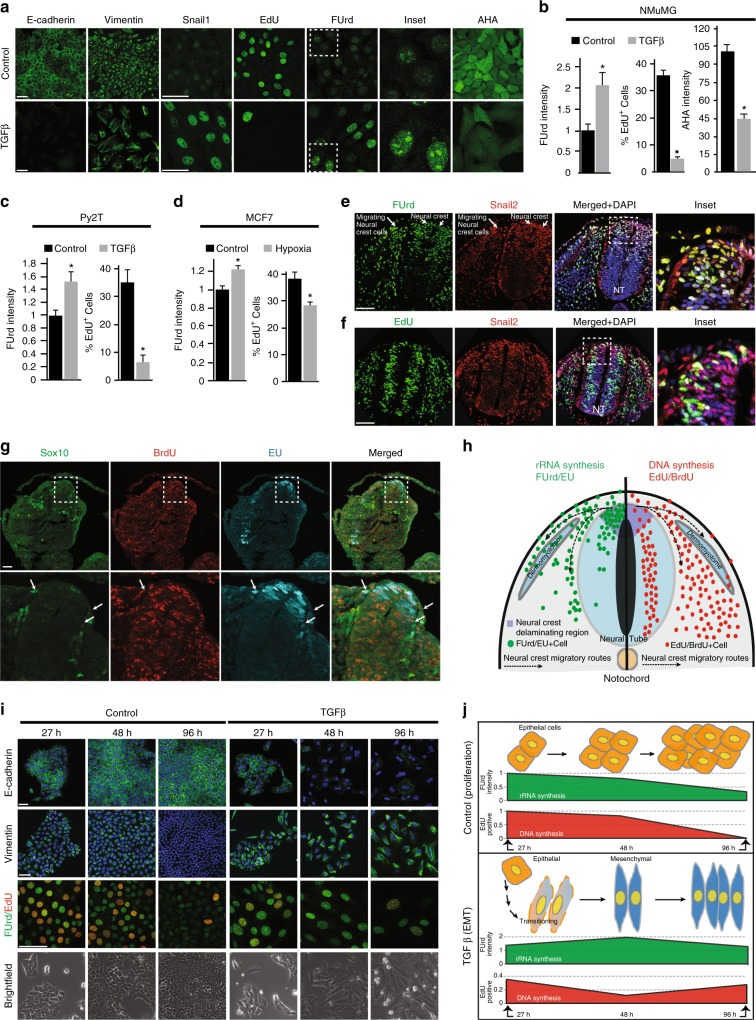
Enhanced rRNA synthesis during EMT is independent of cell proliferation. **a** Proliferating (Control) and 48 h TGFβ-treated (TGFβ) NMuMG cells immunostained for E-cadherin, Vimentin, Snail1, DNA synthesis (EdU), rRNA synthesis (FUrd), and nascent peptide synthesis (AHA) (green). **b** FUrd, EdU, and AHA quantifications from **a**, *P* < 0.01, *P* <  0.001, *P* < 0.05. **c** Quantifications of FUrd and EdU in Py2T cells ± TGFβ, *P* < 0.04, *P*  < 0.04. **d** Quantifications of FUrd and EdU in MCF7 cells ± hypoxia-induced EMT, *P* < 0.001, *P <* 0.05. **e** Immunostaining of FUrd (green), Snail2 (red), and DAPI (blue) in delaminating/migrating neural crest cells (white arrows) of the chick neural tube. Representative inset of the neural crest delaminating region is included. **f** Immunostaining of EdU (green), Snail2 (red), and DAPI (blue) in the chick neural tube with a representative inset of the neural crest delaminating region. **g** Immunostaining of Sox10 (green), DNA synthesis (BrdU, red), and rRNA synthesis (EU, cyan) in mouse E9.0 neural tube. Migrating neural crest cells are indicated with white arrows. **h** Illustration of neural crest delamination/migration showing patterns of detected rRNA (green, left half) and DNA synthesis (red, right half). **i** Immunostaining of E-cadherin (green), Vimentin (green), colocalization of RNA synthesis (FUrd, green) with DNA synthesis (EdU, red), and brightfield images at 27, 48, 96 h ± TGFβ in NMuMG cells. **j** Illustration of quantified rRNA/DNA synthesis (FUrd/EdU) time course from (**i**) in Control (proliferation) and TGFβ (EMT) conditions. Red and green shapes depict FUrd (*P <* 0.01) and EdU (*P* < 0.02) quantifications. All error bars ± SE, *n* = 3. Asterisk denotes *t*-test significance at *P* ≤ 0.05. Scale bar for all images = 50 µm

To investigate rRNA synthesis during EMT, we initially employed the NMuMG cell line, a well-established EMT model system where epithelial cells transition to a mesenchymal state within 48 h of TGFβ treatment^25^. As expected^25^, at 48 h TGFβ-induced NMuMG cells displayed reduced expression of the epithelial marker E-cadherin (Cdh1), as well as coxsackie and adenovirus receptor (Cxadr or CAR) proteins (Fig. 1a; Supplementary Fig. 1a–c). They also exhibited elevated expression of the mesenchymal proteins N-cadherin (Cdh2) and Vimentin (Vim) (Fig. 1a; Supplementary Fig. 1a–c), increased stress fiber formation (Phalloidin staining) (Supplementary Fig. 1a), as well as increased transcription and nuclear localization of the EMT transcription factors Snail1, Smad4, and Twist (Fig. 1a; Supplementary Fig. 1a, c). In line with the previous literature^7,8^, parallel pulse treatment with 5-ethynyl-2′-deoxyuridine (EdU), an established marker of DNA synthesis^26^, showed TGFβ treatment to reduce cellular proliferation by 7-fold (Fig. 1a, b). The non-proliferative status of NMuMG cells in the mesenchymal state was further evidenced by reduced cell division and reduced expression of the proliferation marker Ki67^27^ (Supplementary Fig. 1d–f).

To assess nascent RNA synthesis in epithelial and mesenchymal cell populations, the majority of which is rRNA^28,29^, we briefly pulsed proliferating (Control) and TGFβ-treated (48 h) NMuMG cells with 5-Fluorouridine (FUrd) (Methods). These experiments revealed that FUrd levels were >2 fold higher in the TGFβ-treated cell population and highly localized to the nucleolus (Fig. 1a, b). Cells exhibiting increased nucleolar FUrd staining were generally non-overlapping with those incorporating EdU (Supplementary Fig. 1g, h), consistent with TGFβ-induced rRNA synthesis being independent of cell proliferation. While specific mesenchymal proteins increased in abundance upon TGFβ treatment (Fig. 1a; Supplementary Fig. 1a, b), the induction of rRNA synthesis during EMT was concomitant with a modest decrease in global protein synthesis (Fig. 1a, b; Methods).

To substantiate the finding that EMT is accompanied by rRNA synthesis during a halt in proliferation, we examined the Py2T mammary cell line derived from the MMTV-PyMT mouse model, which also undergoes EMT within 48 h of TGFβ stimulation^30^. Confirming their transition to a mesenchymal state, TGFβ-treated Py2T cells (48 h) displayed significant reductions of E-cadherin and CAR levels, increased Vimentin and Snail1 abundance, and enhanced stress fiber formation (Supplementary Fig. 1i). Mesenchymal Py2T cells also exhibited a concomitant increase in nucleolar FUrd incorporation together with marked reductions in EdU incorporation and Ki67 expression (Fig. 1c; Supplementary Fig. 1j).

To exclude the possibility that these observations were unique to TGFβ-mediated EMT in mouse cells, we employed the human MCF7 breast cancer cell line, which undergoes hypoxia-induced EMT via Notch signalling^31^. MCF7 cells grown for 48 h under hypoxic conditions exhibited both decreased E-cadherin and increased nuclear Snail1 abundance (Supplementary Fig. 1k), characteristic of a mesenchymal state. As observed for both NMuMG and Py2T model systems, hypoxic MCF7 cultures also exhibited increased nucleolar FUrd incorporation and a reduction in the number of EdU^+^ cells (Fig. 1d; Supplementary Fig. 1l).

To corroborate these findings in vivo, we investigated the extent of rRNA and DNA synthesis during the delamination and migration of neural crest cells in chick and mouse embryonic development^8^. Neural crest cells are multipotent progenitor cells that undergo Wnt-driven EMT to facilitate migration from the dorsal neural tube to distinct regions throughout the embryo where they differentiate to epidermal, skeletal, nervous, and connective tissues^8^. To visualize nascent rRNA and DNA synthesis in the chick, embryos were pulsed with FUrd and EdU during early stages of neural crest migration (at Hamburger and Hamilton stage 18/19; Methods). Consistent with an upregulation of rRNA synthesis, delaminating and migrating neural crest cells—identified by positive Snai2 (Snail2) staining^8,32^—exhibited increased nuclear FUrd incorporation, particularly along neural crest migratory routes (Fig. 1e). By contrast, Snail2 expression was largely absent in non-migrating, proliferating (EdU^+^) cells throughout the neural tube (Fig. 1f).

Mouse embryos similarly pulsed with ethynyl uridine (EU; rRNA synthesis^33^) and 5-bromo-2′-deoxyuridine (BrdU; DNA synthesis) at embryonic day 9.0 (E9.0) (Methods), showed that cells exhibiting high-levels of rRNA synthesis were non-overlapping with those exhibiting high-levels of DNA synthesis (Fig. 1g). Delaminating neural crest cells within the dorsal neural tube had higher levels of rRNA synthesis than migratory neural crest cells (defined by the expression of transcription factor Sox10^34^) (Fig. 1g). These findings collectively indicate that increased rRNA synthesis is a general hallmark of the EMT program (Fig. 1h).

### rRNA synthesis induction parallels EMT execution

To examine whether increased rRNA synthesis coincides with execution of the EMT program, or if it is a feature of the mesenchymal end state, we examined rRNA synthesis in NMuMG cells 27, 48 and 96 h after TGFβ treatment (Fig. 1i, j; Supplementary Fig. 1m). After 27 h, Vimentin abundance was increased compared to proliferating (Control) cells, while E-cadherin levels remained unchanged (Fig. 1i; Supplementary Fig. 1n). These findings suggest that execution of the EMT program has only partially completed at this time point. Concomitantly, NMuMG cells displayed decreased DNA synthesis and increased rRNA synthesis (Fig. 1i, j; Supplementary Fig. 1m). We conclude from these data that the induction of rRNA synthesis is closely timed with onset of the EMT program.

At 48 h of TGFβ treatment, EdU incorporation reached a minimum, which was partially regained after 96 h; FUrd incorporation peaked at 48 h and then declined at 96 h (Fig. 1i, j; Supplementary Fig. 1m). Confirming continuation of the EMT program, E-cadherin expression progressively decreased while Vimentin expression further increased (Fig. 1i; Supplementary Fig. 1n). Both EdU and FUrd incorporation decreased over time in proliferating cells, paralleling increased cell confluence (Fig. 1i, j; Supplementary Fig. 1m). We therefore conclude that the EMT program is hallmarked by a divergence in rRNA and DNA synthesis, where rRNA synthesis is transiently induced while DNA synthesis is halted. After EMT is complete, mesenchymal cells then reduce rRNA synthesis to resume proliferation (Fig. 1j).

### EMT is accompanied by increased ribosome biogenesis

**Fig. 2:**
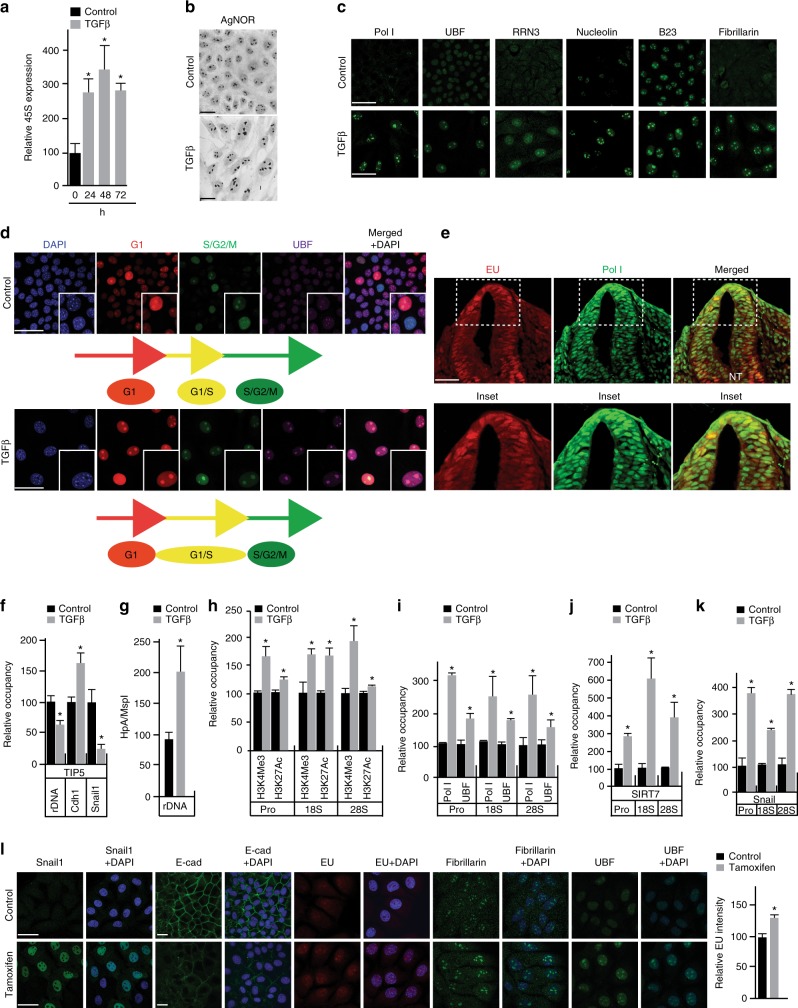
Characteristic features of the EMT-associated ribosome biogenesis program. Cell culture experiments in **b**–**k** depict proliferating (Control) and TGFβ-treated (TGFβ) NMuMG cells at 48 h assessing changes with treatment. **a** qRT-PCR of 45S (pre)-rRNA transcript at 24, 48 and 72 hrs (h), *P* < 0.015. **b** Silver staining of nucleolar organizer regions (NORs). **c** Immunostaining of Pol I, UBF, RRN3, Nucleolin, B23, and Fibrillarin (all green). **d** Cell cycle analysis of proliferating and TGFβ-treated NMuMG cells using the FUCCI technology, DAPI (blue), S/G2/M (geminin, green) and G1 (Cdt1, red), UBF (magenta) and merged. Colocalized green and red fluorescence indicates G1/S arrest (yellow). **e** rRNA synthesis (EU, red), Pol I (green) and merged (yellow) in the mouse neural crest. NT = neural tube, highlighted by white dotted box. **f** ChIP analysis of TIP5 binding to the rDNA, E-cadherin (Cdh1) and Snail1 promoters, *P* < 0.03. **g** HpaII methylation assay of the rDNA promoter, *P* < 0.007. **h** ChIP analysis of H3K4Me3 and H3K27Ac marks to the rDNA promoter, 18S rDNA and 28S rDNA, *P* < 0.006 and *P* < 0.005. **i** ChIP analysis of Pol I and UBF binding to rDNA promoter, 18S rDNA and 28S rDNA, *P* < 0.015 and *P* < 0.04. **j** ChIP analysis of SIRT7 binding to rDNA promoter, 18S rDNA and 28S rDNA, *P* < 0.005. **k** ChIP analysis of Snail1 binding to rDNA promoter, 18S rDNA, and 28S rDNA, *P* < 0.03. **l** Snail1 (green), E-cad (green), EU (red). Fibrillarin (green), UBF (green) and DAPI (blue) in proliferating (Control) and inducible Snail1 overexpression in NMuMG cells. Quantification of EU, *P* < 0.0002. All error bars ± SD, *n* = 3. Asterisk denotes *t*-test significance. Scale bar for all images = 50 µm

Supporting the notion that the induction of rRNA synthesis during EMT is concomitant with increased ribosome biogenesis, 45S pre-rRNA transcript levels reached the highest point in NMuMG cells after 48 h of TGFβ treatment (Fig. 2a). The observed increase in 45S rRNA expression levels correlated with NOR sizes, which were greater than those observed in proliferating (Control) cells (Fig. 2b). Elevated ribosome biogenesis at 48 h was further confirmed by the induction of 34S, 28S, 18S and 5.8S processed rRNA transcripts (Supplementary Fig. 2a–d). As expected for active ribosome biogenesis^15,35^, the increases in rRNA processing observed were concomitant with an increase in the mRNA and protein expression levels of core components of the Pol I transcriptional machinery, including Pol I subunits, UBF, the RNA Pol I-specific transcription initiation factor, RRN3 (Rrn3), the ribosome biogenesis-associated proteins Nucleolin (Ncl) and B23 (Npm1), the 45S processing factor and the rRNA methyltransferase Fibrillarin (Fbl)_,_ and the Pol I-activating NAD-dependent histone deacetylase Sirtuin 7 (SIRT7) (Fig. 2c; Supplementary Fig. 2e–g).

### Ribosome biogenesis in EMT occurs during cell cycle arrest

As cell cycle arrest has been reported to accompany EMT initiation and execution^7,8^, we used FUCCI technologies^36^ to investigate the relationship between the observed increase in rRNA synthesis and cell cycle regulation. As anticipated^37^, after 48 h of TGFβ treatment, NMuMG cells were found to arrest and synchronize at the G1/S transition (Fig. 2d). In line with increased rRNA synthesis, they also displayed enlarged UBF-marked nucleoli compared to proliferating cells (Fig. 2d, inset). Corroborating arrest at the G1/S transition, we simultaneously observed a global decrease in cyclin D1 levels as well as increased levels of nuclear cyclin E (Supplementary Fig. 2h), which together coordinate S phase entry^38^. Evidence supporting increased ribosome biogenesis and cell cycle arrest coincident with the EMT program was also found in TGFβ-induced Py2T cells and hypoxia-induced MCF7 cells (Supplementary Fig. 2i, j) as well as in delaminating, migrating neural crest cell populations in mouse^8^ (Fig. 2e). We conclude from these findings that the level of ribosome biogenesis that accompanies EMT during cell cycle arrest at the G1/S transition is greater than that which normally occurs at this stage of the cell cycle.

### The EMT program activates normally silenced rDNA operons

Mammalian cells possess hundreds of highly homologous, tandemly repeated, rDNA operons^39^. The precise sequences of these rDNA operons are not known and it has yet to be determined if, or how, the distinct mammalian rRNA alleles are differentially regulated in response to physiological stimuli^39^. A significant portion of rDNA operons are silenced through NoRC-regulated heterochromatin formation to ensure nucleolar integrity and genomic stability^21^. NoRC promotes transcriptional downregulation by actively recruiting DNA methyltransferases to mediate epigenetic silencing^21^. Regulated changes in rRNA synthesis can, therefore, be achieved by increasing the rate of transcription from already activated rDNA operons or by decreasing the number that are repressed^21^.

To determine whether the increase in ribosome biogenesis accompanying EMT reflects the activation of rDNA heterochromatin, we examined the expression and localization of TIP5^21^ in proliferating and TGFβ-treated NMuMG cells. Western blot analysis showed a global increase in nuclear TIP5 levels following 48 h of TGFβ treatment (Supplementary Fig. 2k). Chromatin immunoprecipitation (ChIP) experiments revealed, however, that such changes were accompanied by reduced TIP5 association with rDNA promoters (Fig. 2f). Interestingly, and in line with altered E-cadherin and Snail1 expression levels during EMT (Fig. 1a; Supplementary Fig. 2a–c), ChIP analysis revealed reduced TIP5 association with the Snail1 promoter while TIP5 association increased at the E-cadherin promoter (Cdh1) (Fig. 2f).

Corroborating the release of NoRC from rDNA during EMT, TGFβ treatment significantly reduced rDNA promoter methylation (Fig. 2g). These changes were concomitant with an induction of H3K4Me3 and H3K27Ac epigenetic marks, which are canonically associated with actively transcribed genes^21,40^, at rDNA promoters and both 28S and 18S rRNA genes (Fig. 2h). These findings indicate that the relocalization of NoRC during EMT contributes to the transcriptional regulation of Pol I-mediated gene expression. ChIP studies further confirmed an increased recruitment of the core components of Pol I transcription machinery, including Pol I, UBF and SIRT7, to rDNA promoter regions and the 18S and 28S rRNA genes (Fig. 2i, j), consistent with the activation of rRNA expression. We, therefore, conclude that the increase in ribosome biogenesis accompanying EMT is associated with an opening of rDNA operons that are at least partially silenced in proliferating cells, which represent the differentiated, epithelial state.

### Snail1 regulates rRNA synthesis during EMT

Concomitant with Snail1’s established recruitment to the E-cadherin promoter^3,25^ (Supplementary Fig. 2l), ChIP experiments unexpectedly revealed that Snail1 is also recruited to rDNA repeats in a TGFβ-dependent manner (Fig. 2l). To assess whether Snail1 regulates rRNA synthesis, Snail1 levels were induced in NMuMG-Snail1-ERT2 cells by the addition of 4-hydroxytamoxifen^41^ (Methods). In this system, Snail1 induction resulted in a partial EMT, indicated by modest reduction in E-cadherin expression and modest, but significant, increases in rRNA synthesis, nucleolar UBF, and Fibrillarin staining (Fig. 2l). These findings demonstrate that Snail1 contributes to the regulation of rRNA synthesis and ribosome biogenesis during EMT.

### Inhibition of ribosome biogenesis halts EMT

To specifically examine whether ribosome biogenesis is required for cells to transition from an epithelial to a mesenchymal state, we sought to abruptly attenuate rRNA synthesis during EMT by pharmacological means^22^. To do so, we employed CX-5461, an established small-molecule inhibitor of Pol I complex assembly at rDNA promoters, and thus the initiation of ribosome biogenesis^22,24,35,42^.

**Fig. 3:**
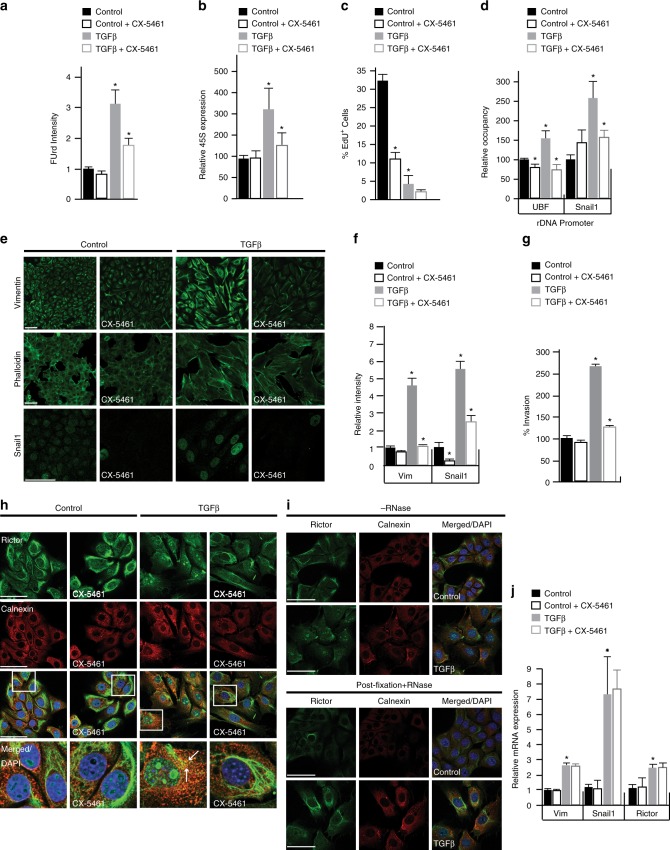
Inhibition of Pol I transcription initiation at rDNA operons impairs the EMT program. All cell culture experiments performed in NMuMG cells ± TGFβ, ± CX-5461 treatments labeled in each panel. **a** Quantification of FUrd ± TGFβ and ± CX-5461; *P* < 0.003. **b** qRT-PCR of 45S (pre)-rRNA transcript ± TGFβ ± CX-5461: Control vs. TGFβ, *P* < 0.05; TGFβ vs. TGFβ  + CX-5461, *P* < 0.02. **c** Quantification of EdU ± TGFβ ± CX-5461, *P* < 0.002. **d** ChIP analysis of UBF and Snail1 binding to the rDNA promoter ± TGFβ ± CX-5461: UBF, *P* < 0.027; Snail1, *P* < 0.018. **e** Immunostaining of Vimentin (green), Phalloidin (green) and Snail1 (green) ± TGFβ ± CX-5461. **f** Quantification of Vimentin (Vim) and Snail1 immunofluorescence from **e**, *P* < 0.02. **g** Relative percent invasion from Boyden chamber invasion assay ± TGFβ ± CX-5461, *P* < 0.002. **h** Immunostaining of Rictor (green), Calnexin (red), Rictor/Calnexin (white arrows, yellow) and DAPI (blue) ± TGFβ ± CX-5461. **i** Immunostaining of Rictor (green) ± RNase A treatment. **j** qRT-PCR of Vimentin (Vim), Snail1 and Rictor mRNA expression, *P* < 0.02. Asterisk denotes *t*-test significance. Error bars ± SE, *n* = 3. Scale bar for all images = 50 µm

For these experiments, we chose a CX-5641 concentration (100 nM) that had little to no impact on rRNA synthesis and ribosome biogenesis in proliferating cells, as measured by FUrd incorporation and 45S rRNA levels (Fig. 3a, b). This concentration is an order of magnitude lower than what has been previously used to block ribosome biogenesis and DNA synthesis in proliferating cells, which induces nucleolar stress, increases nuclear p53 levels and arrests cells in G1 and G2/M^22–24^.

Notably, the administration of CX-5461 (100 nM) to TGFβ-treated NMuMG cells (27 h) significantly reduced FUrd incorporation and 45S pre-rRNA transcription (Fig. 3a, b). In this setting, we observed little to no change in the already suppressed levels of EdU incorporation (Fig. 3c), consistent with TGFβ-treated cells already exhibiting a cessation of DNA synthesis at the time point of CX-5461 administration (27 h) (Fig. 1i, j; Supplementary Fig. 1m). We also observed no effect on nuclear p53 levels (Supplementary Fig. 3a), a canonical marker for nucleolar stress, cell cycle (Supplementary Fig. 3b), or nucleolar UBF localization (Supplementary Fig. 3b). By contrast, the DNA damaging agent, aphidicolin^43^ (APH; 10 µM), reduced DNA synthesis but had no measurable impacts on rRNA synthesis or Snail1 levels (Supplementary Fig. 3c). CX-5461 also exerted only modest impacts on γH2X levels, a readout of both ATM/ATR signaling and DNA damage^24,42^ (Supplementary Fig. 3c).

ChIP studies further revealed that CX-5461 blocked the TGFβ-induced association of UBF and Snail1 with rDNA (Fig. 3d). CX-5461 also significantly reduced the abundance of key mesenchymal markers known to promote the EMT program, including Vimentin, Snail1, and stress fibers, as well as significantly reduce the migratory and invasive capacities of TGFβ-treated cells (Fig. 3e–g). At the same time, CX-5461 had no significant impacts on E-cadherin mRNA or protein levels (Supplementary Fig. 3d), while modestly lowering TGFβ-induced impacts on apoptosis and autophagy (Supplementary Fig. 3e, f). Hence, in the context of TGFβ-mediated cell cycle arrest, CX-5461 (100 nM) mediates the inhibition of ribosome biogenesis to specifically halt the gain of mesenchymal traits associated with EMT, while having no measurable impacts on nucleolar integrity or stress.

Effects of a globally similar nature were also observed when analogous experiments were performed on TGFβ-treated cells using low doses of actinomycin D (Act D; 0.01 µg/mL), which selectively inhibits the elongation phase of Pol I-mediated rRNA synthesis and ribosome biogenesis at low concentrations^24^. Act D did, however, exhibit distinctions from CX-5461 in regards to its more pronounced reductions of rRNA and DNA synthesis and cell invasion in both proliferating and TGFβ-treated cells (Supplementary Fig. 3g, h). Act D also had no significant impact on Vimentin protein levels in the TFGβ context (Supplementary Fig. 3h). These distinctions may relate to Act D’s unique mode of ribosome biogenesis inhibition, which disrupts active Pol I transcription.

To further verify the role of ribosome biogenesis during EMT, we partially silenced the large subunit of Pol I (Polr1a) using RNAi (Methods). Although this approach compromised cell viability, this experiment confirmed that genetic depletion of Pol I during TGFβ treatment reduced EMT, as measured by reduced Vimentin expression (Supplementary Fig. 3i) and invasion (Supplementary Fig. 3j). These results are consistent with ribosome biogenesis being required for execution of the EMT program.

### The impacts of CX-5461 on EMT-associated ribosome biogenesis

Consistent with CX-5461ʼs distinct impacts on proliferating and TGFβ-treated NMuMG cells, gene expression analyses on the actively translating ribosome pool (Methods) revealed that CX-5461-regulated genes were predominantly (~80%) non-overlapping between proliferating and TGFβ-treated cells (Supplementary Fig. 3k). Gene Ontology (GO) analysis^44^ further revealed that the subset of genes commonly upregulated by CX-5461 were enriched for those involved in translation (3.6e−18), while commonly downregulated genes were not enriched for any annotated GO category. Hence, CX-5461 has distinct impacts on gene expression in proliferating and TGFβ-treated cells although ribosome biogenesis is a common denominator in both systems.

### The EMT-associated ribosome biogenesis is linked to Rictor

mTORC2 is a key driver of EMT^45^, whose activation is mediated through ribosome association^13,14^. Given the observed necessity of ribosomal biogenesis for execution of the EMT program, we investigated the potential connection between rRNA synthesis and mTORC2 signaling through the assessment of the Rictor component of mTORC2^13,46^. As expected^13^, prior to the treatment of NMuMG cells with TGFβ, Rictor predominantly localized to filamentous structures in the cytoplasm and to the endoplasmic reticulum (ER), indicated by its colocalization with the ER marker Calnexin (Fig. 3h). Consistent with mTORC2 activation during EMT^45^, Rictor exhibited a pronounced increase in ER localization following 48 h of TGFβ treatment (Fig. 3h).

Strikingly, Rictor also showed a pronounced increase in nucleolar localization upon TGFβ treatment (Fig. 3h), which was specifically abolished by RNase treatment prior or subsequent to fixation (Fig. 3i, Supplementary Fig. 3l). Consistent with its association with nascent rRNA transcripts, Rictor localization to nucleoli was reduced by CX-5461 administration (100 nM) after 27 hrs of TGFβ treatment (Fig. 3h, Supplementary Fig. 3m). In line with reduction in mTORC2 signaling and disruption of the EMT program^45^, CX-5461 also diminished Rictor’s association with the ER (Fig. 3h). Notably, CX-5461 exerted no changes on the mRNA transcript levels of Rictor or the mTORC2-regulated mesenchymal markers Vimentin and Snail1 (Fig. 3j). Smad4 expression also remained unchanged (Supplementary Fig. 3n).

### Pol I and Rictor levels increase during tumor progression

**Fig. 4:**
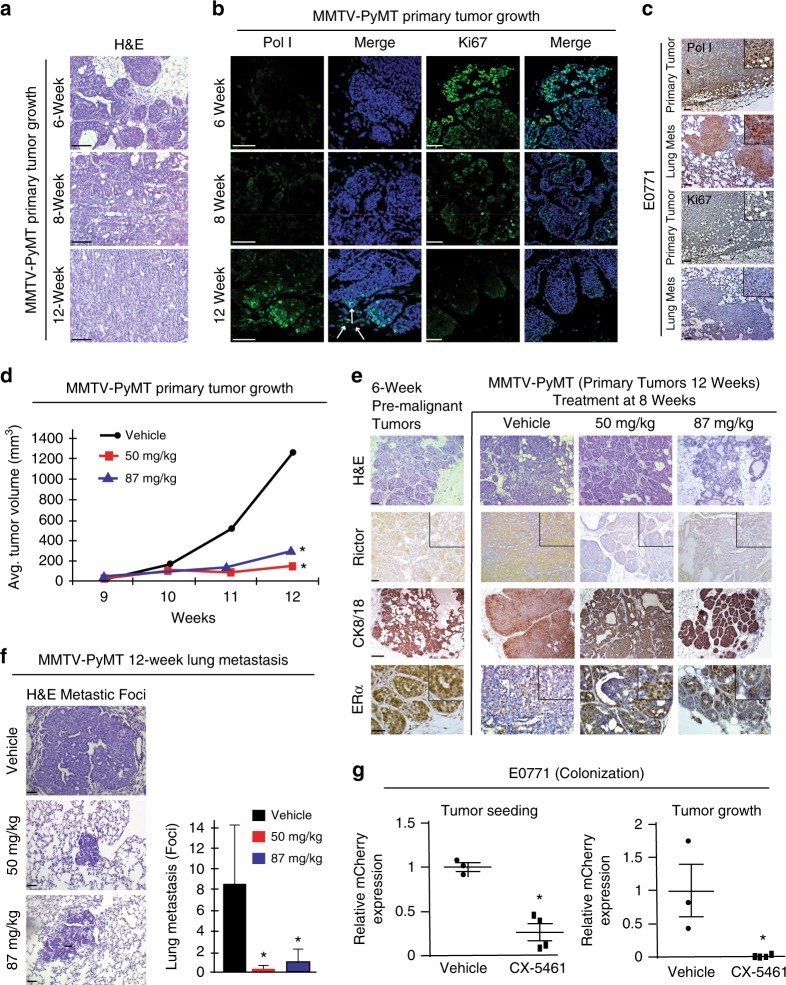
Ribosome biogenesis inhibition reduces primary tumor growth and metastasis. **a** H&E staining of 6-, 8- and 12-week MMTV-PyMT mouse primary tumor samples. Scale bar = 240 µm. **b** Immunostaining for Pol I (green) and Ki67 (green) merged with DAPI (blue) in MMTV-PyMT mouse tumors at 6-, 8- and 12-week. White arrows = tumor front. Scale bar = 50 µm. **c** IHC staining for Pol I and Ki67 in E0771 primary tumor and corresponding lung metastasis. Scale bar = 240 µm. **d** Quantification of MMTV-PyMT primary tumors: vehicle (PBS), 50 mg/kg, and 87 mg/kg of CX-5461, ANOVA *P* < 0.01, *n* = 4 vehicle, *n* = 3 per CX-5461 group. **e** H&E staining of MMTV-PyMT primary tumors: vehicle-treated, 50 mg/kg or 87 mg/kg CX-5461 as well as 6-week pre-malignant tumors. IHC staining of Rictor, Cytokeratin 8/18 (CK8/18) and estrogen receptor alpha (ERα) expression in vehicle-treated, 50 mg/kg, or 87 mg/kg CX-5461 tumors or 6-week pre-malignant tumors. Scale bar = 50 µm. **f** H&E staining of metastatic lesions in CX-5461-treated and vehicle-treated mice. Scale bar = 50 µm. Quantification of lung metastasis in CX-5461-treated and vehicle-treated mice, ANOVA *P* < 0.02. Error bars ± SD. **g** Quantification of mCherry positive E0771 cells seeded and colonized in the lungs of vehicle and 50 mg/kg CX-5461-treated C57BL/6 mice, *t*-test *P* < 0.01. Quantification of metastatic growth of mCherry positive E0771 cells in the lungs of vehicle and 50 mg/kg CX-5461-treated C57BL/6 mice, *t*-test, *P* < 0.05. *n* = 3 vehicle, *n* = 4 CX-5461 treatment. All asterisks denote significance

We next examined the relevance of rRNA synthesis to tumor growth and metastasis in the MMTV-PyMT mouse. This model system mimics the development of human luminal breast cancer progression from focal hyperplasia through adenoma into early and late carcinomas with a progressive loss of luminal markers and hormonal receptor expression that metastasize to the lung^47^. Hematoxylin and Eosin (H&E) staining of MMTV-PyMT mouse mammary luminal tumors at 6 weeks showed hyperplastic regions with minimally invasive characteristics (Fig. 4a). At this stage, which is prior to metastasis (Fig. 4a), only low levels of Pol I expression were observed in the hyperplasic areas (Fig. 4b). At 8 weeks of age, areas of the mammary glands displayed adenoma-like tumors and micro-metastases were also detected in the lung (Fig. 4a; Supplementary Fig. 4a). By 12 weeks, basal carcinoma-like tumors, typical of a poor prognostic outcome, were detected with highly invasive characteristics, including extensive stromal and immune cell infiltration^47^ (Fig. 4a). At 8- and 12-week assessment points, we observed prominent staining of Pol I that was notably enhanced at invasive tumor fronts (Fig. 4b, white arrows). The observed increase in rRNA synthesis correlated with an increase in Rictor protein levels, as evidenced by immunohistochemical staining (IHC) (Supplementary Fig. 4b). By contrast, Ki67 staining gradually decreased with tumor progression (Fig. 4b), indicating that the observed increased rRNA synthesis was occurring within the largely non-proliferative primary tumor region. At 12 weeks, strong Pol I staining was also observed within secondary lung metastases (Supplementary Fig. 4c). Similar patterns of Pol I and Ki67 levels were also observed in both primary mammary tumors and secondary lung metastases in the basal-like medullary adenocarcinoma E0771 mouse model^48^ (Fig. 4c; Methods). These data corroborate our in vitro findings and indicate that Pol I expression is induced within largely non-proliferative tumor cell populations in distinct mouse models representing distinct subtypes of breast cancer during disease progression.

### Ribosome biogenesis inhibition induces tumor differentiation

To investigate the role of rRNA synthesis in cancer invasion, spread, and metastasis, MMTV-PyMT mice with palpable tumors (8-weeks) were treated weekly with CX-5461 (50 or 87 mg/kg). Consistent with an anti-proliferative impact^22–24,49^, significantly smaller tumor volumes were detected with both doses of CX-5461 treatment over a 4-week time window (8–12 weeks) (Fig. 4d). Histological examination of H&E-stained, CX-5461-treated primary tumors revealed a striking change of morphology indicative of tumor regression and differentiation to a benign phenotype (Fig. 4e). Confirming this interpretation, for both CX-5461 treatment regimens, the levels of the luminal epithelial differentiation marker Cytokeratin 8/18 (CK8/18)^50^ increased and returned to ductal areas, closely resembling the expression pattern observed in pre-metastatic (6 weeks) primary tumors (Fig. 4e). Elevated CK8/18 expression is associated with reduced cell invasion and lung metastasis both in vitro and in vivo^50^. Importantly, ERα expression also increased in CX-5461-treated tumors (Fig. 4e), further confirming tumor differentiation to a non-invasive, luminal phenotype^51^.

Corroborating our in vitro findings (Fig. 3a–c, e, f; Supplementary Fig. 3c), and previous demonstrations that CX-5461 inhibits rRNA synthesis in vivo^22–24,49^, Snail1/2 levels were reduced in the CX-5461-treated mice akin to the expression pattern observed in pre-metastatic (6 weeks) primary tumors (Supplementary Fig. 4d). CX-5461 treatment did not affect autophagy, as indicated by LC3 staining^52^ (Supplementary Fig. 4e). In line with mTORC2’s contribution to ribosome biogenesis-associated tumor progression and dedifferentiation^13,53^, we also observed a marked reduction in Rictor expression following CX-5461 treatment (Fig. 4e; Supplementary Fig. 4b). Given that Snail1 is positively regulated by mTORC2^45^, and that Snail1 suppresses CK8/18 and ERα expression during EMT^45,54,55^, we conclude that CX-5461-mediated inhibition of rRNA biogenesis in vivo induces tumor differentiation through the disruption of an mTORC2/Snail1/ERα signaling axis. Consistent with regression to a benign and less invasive phenotype, both CX-5461 regimens reduced the size and number of lung metastases by roughly 90% (Fig. 4f). Hence, the inhibition of rRNA synthesis by CX-5461, in addition to its antiproliferative effects, can act to induce tumor differentiation and reduce metastasis in vivo.

### Ribosome biogenesis inhibition reduces metastatic seeding

To test whether rRNA synthesis contributes to metastasis in the absence of a primary tumor, metastatic seeding and lung colonization were examined using the syngenic, basal-like, E0771 metastasis mouse model^48^. To do so, C57BL/6 mice were either treated with vehicle or pre-treated with CX-5461 (50 mg/kg) 24 h prior to tail vein injection of mCherry-labeled E0771 cells. Tumor cell injections were then followed by CX-5461 dosing (50 mg/kg) twice per week over a 5-week period. In mice pre-treated with CX-5461, injected tumor cells were less capable of seeding and colonization in the lung (Fig. 4g). Lung metastasis colonization was also significantly decreased when E0771 cells were tail vein injected into mice that were subsequently dosed with CX-5461 (50 mg/kg) 24 h post injection, followed by 2 weeks bi-weekly CX-5461 dosing (50 mg/kg) (Fig. 4g). These data indicate that CX-5461-mediated inhibition of rRNA synthesis in vivo attenuates pro-invasive programs, limiting metastasis through the inhibition of seeding and metastatic tumor growth.

### Pol I expression correlates with dedifferentiation

**Fig. 5:**
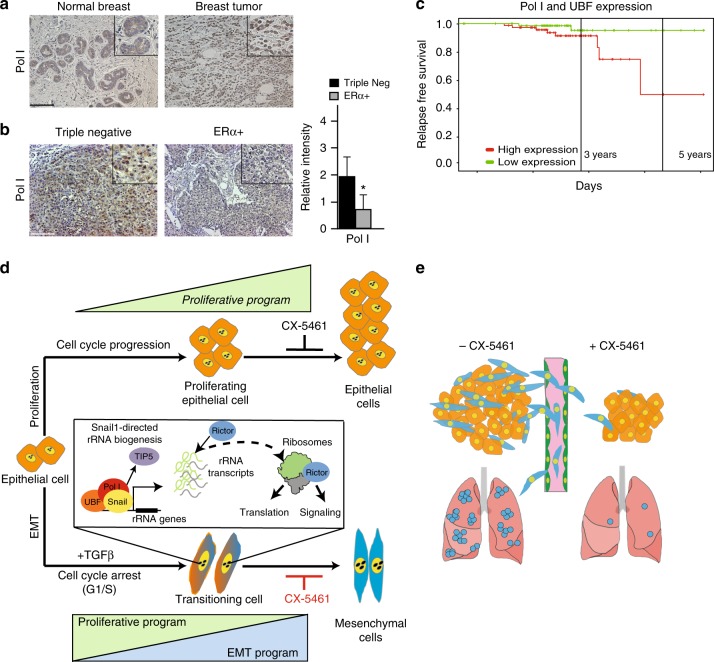
Human breast tumors exhibit high-levels of Pol I expression. **a** IHC staining of Pol I in normal human breast tissue and invasive breast tumor tissue. **b** IHC staining of Pol I in TNBC and ERα^+^ tumors. Pol I intensity scoring TNBC compared to ERα^+^ tumors, *t*-test, *P* < 0.01. Error bars ± SD. Asterisk denotes significance. Scale bar = 240 µm. **c** Survival curve showing induced expression of Pol I and UBF correlates with lower relapse-free survival in patients with breast cancer, *P* < 0.006. **d** Schematic model showing TGFβ-induced, G1/S arrest of the cell cycle during EMT, accompanied by association of Snail1, Pol I and UBF with rDNA operons, TIP5 dissociation, and the generation of new Rictor-associated ribosomes. **e** Model depicting reduced primary tumor growth and metastasis via CX-5461-mediated inhibition of rRNA synthesis

To assess the clinical relevance of our findings, we examined Pol I in normal human breast tissue and in invasive breast tumors. Invasive tumors exhibited significantly higher levels of Pol I staining compared to normal tissues (Fig. 5a). Pol I expression was also more highly expressed in aggressive, dedifferentiated basal-like triple negative breast cancers (TNBC) compared to less aggressive differentiated, luminal ERα^+^ tumors (Fig. 5b). Notably, high Pol I and UBF expression correlates with a reduced probability of relapse-free survival (Fig. 5c). While further experiments are required to delineate the relative contributions of Pol I expression in these systems to proliferation versus EMT, the evidence presented suggests that the EMT-associated ribosome biogenesis program contribute to dedifferentiation and human breast cancer progression.

## Discussion

rDNA transcription, the initiating step in ribosome biogenesis, is canonically associated with an elevation of protein synthesis to accommodate growth and cell division^18,19,29,35^. Unexpectedly, we show that the EMT program, a cell identity switch critical to development, wound healing, and disease^5^ is accompanied by transcriptional activation of rDNA operons that are normally silenced, or partially silenced, in the epithelial state. Notably, we find that this feature of EMT is independent of initiating stimulus and species. Interestingly, the EMT-associated ribosome biogenesis occurs when cells are arrested at the G1/S transition, the “restriction point” prior to the commitment to cell division^38^. These changes may be mediated, at least in part, by increased nuclear Snail1 levels, which transcriptionally represses cyclin D1 expression via direct binding to its promoter region^7^.

During G1/S arrest, we observe that global levels of protein synthesis are modestly reduced compared to proliferating cells, while specific mesenchymal markers that contribute to the cellular capacity to migrate and invade distal tissues are increased. Consistent with this notion, our pharmacological and RNAi-mediated inhibition studies argue that ribosome biogenesis during EMT fuels a gene expression program that is required to achieve the mesenchymal cell state. Our investigations of chick and mouse development show that rRNA synthesis levels are highest in delaminating neural crest cells, where defects in ribosome biogenesis have been associated with severe developmental malformations^56,57^. Roles for ribosome biogenesis and translational control have also been evidenced in congenital malformations linked to neural crest development^56,57^. These findings collectively argue that ribosome biogenesis during cell cycle arrest is a general feature of the EMT program.

Stem cells are permissive to differentiation signals, including TGFβ, during the G1 phase of the cell cycle^58^. Differentiation towards the three embryonic germ layers is canonically linked to heterochromatin decompaction in preparation for DNA replication and the induction of distinct Pol II-driven transcriptional programs^58^. The present investigations suggest that Pol I activities are also regulated during the cell cycle, and cell cycle arrest at the G1/S transition in particular.

A distinguishing feature of the ribosome biogenesis program evidenced during EMT is its significant elevation relative to that which is observed in the G1 phase of normally cycling cells. The EMT-associated ribosome biogenesis program is also distinguished by a marked increase in Snail1 binding to rDNA operons concomitant with release of the repressive NoRC. Notably, increased Snail1 expression alone is sufficient to induce rRNA synthesis in the absence of TGFβ. Reciprocal Snail1 silencing studies during TGFβ-induced EMT, while potentially informative to understanding Snail1-independent aspects of TGFβ signaling, were not considered here due to Snail1’s central role in a multitude of distinct aspects of the EMT program, potentially including cell cycle arrest. Snail’s active participation in the EMT-associated ribosome biogenesis program extends its pervasive Pol II regulatory capacities^3^ to the Pol I machinery, putting it in a similar class as the Myc oncogene as a pan-RNA polymerase regulator^59^.

The distinct nature of the EMT-associated ribosome biogenesis program is further evidenced by the increase in Rictor expression at the point of G1/S arrest and its localization to nucleoli. In proliferating cells, Rictor expression is normally enhanced during S phase^60^ and its localization is primarily cytoplasmic, where it associates with mitochondria-associated endoplasmic reticulum membranes (MAMs)^61^. mTORC2’s contributions to the mesenchymal gene expression program and its activation upon ribosome association are well established^13,14,45^. We speculate that the EMT-associated ribosome biogenesis program, driven by Snail1-directed changes in rDNA transcription, directs Rictor’s association with newly generated ribosomes. In so doing, the EMT-associated ribosome biogenesis program may template mTORC2’s assembly with ribosomes to thereby promote its activation and subsequent regulation of gene expression (Fig. 5e). In support of this “ribo-interactome” model^62,63^, CX-5461-mediated inhibition of ribosome biogenesis during EMT reduces Snail1’s association with rDNA, abrogates Rictor’s nucleolar and MAM localization, and hampers the expression of mTORC2-regulated mesenchymal markers. At the same time, Smad4 localization remains intact, suggesting a Smad4-independent mechanism of Rictor association with ribosomes and mTORC2 activation. We note in this context that EMT can also be blocked by the inhibition of ribosome export from the nucleus^64^. Further experiments will be required to validate these mechanistic connections, including focused investigations into the activation status of downstream mTORC2 signaling cascades. Additional studies will also be needed to delineate the specific signaling pathways contributing to activation of the EMT-associated ribosome biogenesis program in different physiological contexts.

Cells arrested in G1/S during EMT are significantly larger than those that are cycling normally^65^. The induction of ribosome biogenesis during EMT may, therefore, reflect homeostatic mechanisms that scale ribosome concentration in relation to changes in cell size^17,66^. Ribosome biogenesis may, however, also play a more active role in facilitating execution of the EMT program by influencing gene expression and thus cell fate. For instance, the induction of rRNA expression may increase the number of active ribosomes to elicit gene-specific impacts on translation efficiency, including increased protein production from transcripts containing long, structured 5′-untranslated regions^67^. Changes in ribosome abundance may also perturb the translation efficiencies of specific genes by altering the availability of protein synthesis components or other complexes, such as mTORC2, that mediate cellular signaling cascades through association with the ribosome^13,14^. Primary rRNA transcripts and intermediates of ribosome assembly may also sequester cellular factors to indirectly influence protein synthesis^68,69^. Mechanisms wherein newly synthesized ribosomes exhibit physical and functional distinctions, which give rise to gene-specific translational control, can also be envisaged^39,62^. In this context, it will be critical to determine whether the EMT-associated ribosome biogenesis program activates all rDNA operons equally or if there are physiological alterations in the relative expression of specific rDNA operons, as has been observed in other systems^39,70,71^. Distinguishing whether the EMT-associated ribosome biogenesis program reflects the upregulation of specific classes of rDNA operons normally silenced in the differentiated state, and if the upregulation of ribosome biogenesis is a causal feature of the mesenchymal gene expression program, will require deeper knowledge of the full repertoire of rDNA operon sequences encoded by mammalian cells. Detailed investigations of the ribosome pool and the specific translation programs prior and subsequent to EMT may also aid examinations of the relationship between the EMT-associated ribosome biogenesis program and the accompanying changes in gene expression and cell fate.

The importance of such pursuits is highlighted by the pre-clinical studies presented, which reveal that pharmacological inhibition of rRNA synthesis slows primary tumor growth and differentiates tumors in a manner that reduces metastatic seeding and growth (Fig. 5e). Such changes were found to stem, at least in part, from reductions in Rictor and Snail1/2 protein levels. We infer from these findings that programmed changes in the translational capacity of migratory mesenchymal cells, enabled by the EMT-associated ribosome biogenesis program, influence the process of colonization at new tumor growth sites (Fig. 5e).

In this context, it is particularly striking that CX-5461 can induce solid tumor differentiation from a basal-like to a luminal ERα+ phenotype. This finding suggests that CX-5461 may have the potential to restore or enhance endocrine therapy responsiveness in ERα- or endocrine-resistant patients, whose tumors are typically more dedifferentiated, aggressive and prone to reoccurrance and metastasis^72,73^. The capacity of CX-5461 to mediate such impacts represents a rare demonstration that a small-molecule can induce differentiation in solid tumors, which may also afford potential synergies with anti-proliferative therapies. These possibilities motivate focused assessments of the precise mechanisms by which rRNA synthesis influences tumor plasticity, metastatic spread and secondary tumor growth, wherein mesenchymal cells renew their proliferative capacity^3^. Initiatives of this kind must include in-depth explorations of CX-5461’s specific molecular target(s) in the cell, the mechanistic basis of it’s actions and how, specifically, CX-5461-mediated inhibition of rRNA synthesis during EMT regulates the mesenchymal gene expression program. Given the findings presented, such efforts may have the potential to offer new avenues for differentiation therapies^74^ and to inform on new strategies to treat aggressive, metastatic cancers for which current approaches are inadequate.

## Methods

### Cell culture and reagents

Namru Mus musculus mammary gland (NMuMG) cells acquired from ATCC and NMuMG-Snail1-ERT2 (gift from Dr. Nieto) were maintained in Dulbecco’s Modified Eagle Medium (DMEM, Gibco/Invitrogen), 10% fetal bovine serum (FBS, Gibco/Invitrogen), GlutaMAX (Gibco #35050), penicillin–streptomycin (PenStrep, Gibco #15140122) and insulin 10 µg/mL (Sigma#I0516). Michigan Cancer Foundation-7 (MCF7) cells were a gift from Dr. Jonas Fuxe, Karolinska Institute, and Py2T cells were a gift from Prof. Gerhard Christofori, University of Basel. Both were grown in DMEM with 10% FBS, GlutaMAX and PenStrep. The NMuMG-Fucci2 cells (RCB2868) were obtained from the RIKEN BRC through the National Bio-Resource Project of the MEXT, Japan and Dr. Atsushi Miyawaki.

Recombinant human TGFβ1 protein (R&;D, #240B) was added to 10 ng/mL to induce EMT. Snail1 was induced in NMuMG-Snail1-ERT2 cells by the addition of 4-hydroxytamoxifen at 200 nM for 72 h. CX-5461 (Cylene Pharmaceuticals, San Diego, USA, Selleckchem, San Diego, USA) was added to a final concentration of 100 nM, actinomycin D (A1410, Sigma) was added to a final concentration of 0.01 μg/mL and aphidicolin (A0781, Sigma) to a final concentration of 10 µM. For hypoxia, cells were transferred to a hypoxia chamber (1% oxygen) for 48 h prior to analysis. FUrd and EdU chases were performed post-hypoxia under normal culturing conditions. All cell lines were routinely checked for mycoplasma contamination.

### Immunostaining

Cells were plated on glass cover slips at 20% confluency one day prior to treatment with TGFβ (or vehicle). CX-5461 (100 nM), actinomycin D (0.01 μg/mL), or aphidicolin (10 μM) were added 27 h post TGFβ treatment for an additional 24 h. After treatment, depending on the intended primary antibody, cells were fixed with 4% paraformaldehyde, ice-cold ethanol or ice-cold methanol (Supplementary Table 1). Formaldehyde fixation continued for 15 min, followed by permeabilization with 0.3% Triton X-100 in PBS for 15 min and 1 h of blocking with 1% BSA in PBS with 0.3% Triton X-100. Methanol fixation was limited to 20 s and ethanol fixation to 10 min at room temperature. Post ethanol fixation cells were permeabilized for 5 min with 0.1% Triton X-100 in PBS, and samples fixed by both methanol and ethanol were blocked for 1 h with 1% BSA-PBS. See Supplementary Table 1 for antibody details. With formaldehyde fixation, after blocking, cells were stained overnight at 4 °C with primary antibodies diluted in 1% BSA in PBS with 0.3% Triton X-100. The following day, cells were incubated for 1 h with secondary antibodies diluted 1:1000 in 1% BSA in PBST. Cells were washed three times with PBST after antibody incubation. The same antibody incubation and washing procedures were followed for cells fixed with methanol or ethanol, however Triton X-100 was omitted in the wash and antibody incubation steps. For both phalloidin staining and processing of 5-ethynyl-2′-deoxyuridine (EdU) pulsed cells with azide-Click-IT technology, manufacturer’s protocols were followed. Secondary antibodies included Alexa Fluor 647 goat anti-mouse and Alexa Fluor 647 goat anti-rabbit (A21233 and A21244, Invitrogen). Following secondary antibody staining, phalloidin or EdU protocols, cells were washed three times with PBS and stained with DAPI diluted in PBS and mounted on cover slips. Cover slips were visualized using Leica and Zeiss LSM 710 confocal microscopes. Each experiment was performed on 3 biological replicates.

### RNase A treatment protocol

Pre-fixation: Proliferating and TGFβ-treated cells were washed twice with PBS for 3 min before treatment with 2 mg/mL RNase A in PBS for 15 min at room temperature. Cells were fixed with 4% paraformaldehyde in PBS for 15 min at room temperature before proceeding to immunofluorescence staining. Post-fixation: Proliferating and TGFβ-treated cells were washed twice with PBS for 3 min and were fixed with 4% paraformaldehyde in PBS for 15 min at room temperature. The cells were washed twice with PBS for 3 min before treated with 2 mg/mL RNase A in PBS for 15 min at room temperature. The cells were washed twice with PBS before proceeding to immunofluorescence staining. Each experiment was performed on 3 biological replicates.

### Cell count

70,000 NMuMG cells were seeded per well of a 6-well plate. Both proliferating and TGFβ-treated cells were trypsinized after 48 h and counted using the NucleoCounter® NC-3000™. Experiment was performed on 3 biological replicates, error bars are the mean ± SD and the two-tailed Students *t*-test, *P* < 0.001 (Supplementary Fig. 1f).

### Brightfield microscopy

Unfixed cells were imaged using a Zeiss Axiovert 40 CFL microscope using an AxioCam ICm1 camera and Axiovert software.

### Quantifications of immunostaining

Fluorescent signal intensity was quantified using ImageJ software. Images were converted to 8-bit depth, thresholded and signal intensity was quantified by measurement of ‘Integrated Density’ taking into account both signal intensity and area of signal expression. Threshold values were consistent across all treatment conditions for a given marker. The resulting ‘Integrated Density’ value was then divided by the number of cells in the field as identified through DAPI staining. The treatment values are relative to the value of the control condition, which was set to a value of one (1). For EdU and Ki67, the number of EdU^+^ or Ki67^+^ cells were counted, and this number was then divided by the total number of cells in the field, identified by DAPI staining, and converted to a percentage value. Colocalization of FUrd and EdU was determined by measurement as the percentage of FUrd signal that was in EdU positive cells out of total FUrd signal per 100 cells per condition, and graphs shown represents how much of that signal was localized to EdU^+^ cells.

### Statistics for quantification of immunostaining

For all quantification, asterisks denote significance as assessed with two-tailed Student’s *t*-test. In figure legends, the highest *P*-value is shown.

### Statistics for Figure 1 data

Quantification of FUrd incorporation in NMuMG cells calculated as average signal intensity per cell, *P* < 0.01 (Fig. 1b). Quantification of EdU positive cells out of a total number of DAPI-positive cells in NMuMG cells, *P* < 0.001 (Fig. 1b). Quantification of AHA incorporation in NMuMG cells calculated as average signal intensity per cell, *P* < 0.0005 (Fig. 1b). Quantifications of average FUrd signal intensity and of percentage EdU positive cells out of a total number of DAPI-positive cells with and without TGFβ treatment in Py2T cells, *P* < 0.04 (EdU and FUrd) (Fig. 1c). Quantifications of average FUrd signal intensity and percentage EdU positive cells out of a total number of DAPI-positive cells with and without hypoxia-induced EMT in MCF7 cells, *P* < 0.05 (EdU), *P* < 0.001 (FUrd) (Fig. 1d). Quantification of signal during time course ± mean standard error: FUrd control 27 h (0.04), 48 h (0.08) and 96 h (0.05); TGFβ-treated 27 h (0.06), 48 h (0.15) and 96 h (0.26); EdU control 27 h (0.007), 48 h (0.01) and 96 h (0.01); TGFβ-treated 27 h (0.14), 48 h (0.05) and 96 h (0.14) (Fig. 1i, j). Assessment of time course via Student’s *t*-test: FUrd, control compared to TGFβ (27, 48 and 96 h) and control (96 h) compared to control (48  and 27 h), *P* < 0.01; EdU: control (27 h) compared to control (48  and 96 h), and control compared to TGFβ (27, 48 and 96 h), *P* < 0.02 (Fig. 1i, j).

### Statistics for Supplementary Figure 1 data

Quantification of Ki67 positive cells out of a total number of DAPI positive cells in NMuMG cells, *P* < 0.001 (Supplementary Fig. 1e). Quantification of localization of FUrd signal to EdU positive cells with and without TGFβ treatment, *P* < 0.001 (Supplementary Fig. 1h). Quantification of time course (Fig. 1i; Supplementary Fig. 1n), Vimentin and E-cadherin at 27, 48 and 96 h in control and TGFβ treated NMuMG cells. E-cadherin: control compared to TGFβ (48 and 96 h), *P* < 0.002; between TGFβ time points, *P* < 0.05. Vimentin: control compared to TGFβ (27, 48 and 96 h), *P* < 0.02; control (48 h) compared to control (96 h) and for all comparisons of TGFβ conditions, *P* < 0.02.

### Statistics for Figure 2 data

Quantification of EU incorporation in NMuMG cells calculated as average signal intensity, *P* < 0.0002 (Fig. 2l).

### Statistics for Figure 3 data

Quantification of average FUrd signal intensity per cell and percentage of EdU^+^ cells in control, CX-5461, TGFβ, and TGFβ+CX-5461 treated NMuMG cells (Fig. 3a, c). FUrd: *P* < 0.003 control compared to TGFβ, and TGFβ compared to TGFβ+CX-5461. EdU; *P* < 0.002, control compared to TGFβ, and control compared to control+CX-5461. Quantification of Vimentin and Snail1 immunofluorescence intensity in control, CX-5461-treated, TGFβ and TGFβ+CX-5461-treated NMuMG cells (Fig. 3f). Vimentin: control compared to TGFβ and TGFβ compared to TGFβ+CX-5461, *P* < 0.001. Snail1: control compared to TGFβ and TGFβ compared to TGFβ+CX-5461, *P* < 0.002; control compared to CX-5461, *P* < 0.02.

### Statistics for Supplementary Figure 3 data

Quantification of p53 immunofluorescence intensity in control, CX-5461-treated, TGFβ-and TGFβ + CX-5461-treated NMuMG cells, *P* < 0.001 (Supplementary Fig. 3a). Quantification of UBF immunofluorescence intensity in control, CX-5461-treated, TGFβ- and TGFβ + CX-5461-treated NMuMG cells, *P* < 0.001 (Supplementary Fig. 3b). Quantification of average FUrd signal intensity per cell and percentage of EdU^+^ cells in control, actinomycin D-, TGFβ- and TGFβ + actinomycin D-treated NMuMG cells (Supplementary Fig. 3g). FUrd: control compared to control + actinomycin D, *P* < 0.02; control compared to TGFβ, and TGFβ compared to TGFβ + actinomycin D, *P* < 0.001. EdU: control compared to actinomycin D and control compared to TGFβ, *P* < 0.001. Quantification of Vimentin and Snail1 immunofluorescence intensity in control, actinomycin D-treated, TGFβ- and TGFβ + actinomycin D-treated NMuMG cells (Supplementary Fig. 3g). Vimentin: control compared to actinomycin D, *P* < 0.01; control compared to TGFβ, *P* < 0.01. Snail1: control compared to TGFβ and TGFβ compared to TGFβ + actinomycin D, *P* < 0.002 (Supplementary Fig. 3h). Quantification of percentage of EdU^+^ cells in control, aphidicolin-, TGFβ- and TGFβ + aphidicolin-treated NMuMG cells, *P* < 0.001(Supplementary Fig. 3c). Quantification of average Pol I, EU and Vimentin signal intensity per cell in ctrl siRNA and Pol1 siRNA treated NMuMG cells, *P* < 0.0002 (Supplementary Fig. 3i). Quantification of average FUrd signal intensity per cell, aphidicolin-, TGFβ- and TGFβ + aphidicolin-treated NMuMG cells. FUrd: control compared to TGFβ, *P* < 0.002 (Supplementary Fig. 3c). Quantification of Snail1 immunofluorescence intensity in control-, aphidicolin-, TGFβ- and TGFβ + aphidicolin-treated NMuMG cells, *P* < 0.01 (Supplementary Fig. 3c). Quantification of Rictor immunofluorescence intensity in control, CX-5461-treated, TGFβ- and TGFβ + CX-5461-treated NMuMG cells. Rictor: control compared to TGFβ and TGFβ compared to TGFβ + CX-5461, *P* < 0.0001; control compared to CX-5461, *P* < 0.0001 (Supplementary Fig. 3m). Quantification of Smad4 immunofluorescence intensity in control, CX-5461-treated, TGFβ- and TGFβ + CX-5461-treated treated NMuMG cells, *P* < 0.001 (Supplementary Fig. 3n)

### Western blotting

Cells were lysed and sonicated in RIPA buffer (50 mM Tris-HCl pH 7.5, 150 mM NaCl, 1 mM EDTA, 1% NP40, 0.5% sodium deoxycholate, 0.1% SDS) supplemented with protease inhibitors (cOmplete cocktail EDTA-free, Roche). Protein extracts were boiled in sample buffer (BioRad), separated by SDS–PAGE under reducing conditions and transferred to nitrocellulose filters (BioRad) by semi-dry electro-blotting. Nuclear fractions for TIP5 were obtained using NE-PER cell fractionation kit (Thermo Scientific, #78833). Primary antibodies are listed in Supplementary Table 1. Immunoreactive bands were visualized by chemi-luminescence (BioRad) and a BioRad ChemiDoc XRS imaging system. Each experiment was performed on 3 biological replicates.

### rRNA, DNA, and nascent peptide synthesis in vitro

FUrd assay was performed as previously described^28,29^. Cells were pulsed with 2 mM FUrd for 8–10 min following 48 h of treatment (TGFβ or hypoxia), under normal culturing conditions. Following the pulse, cells were rinsed with PBS and fixed with paraformaldehyde. In addition, cells were pulsed with 20 μM EdU for 45 min according to manufacturer instructions under normal culturing conditions, following the pulse cells were rinsed with PBS and fixed with paraformaldehyde. Click-it assay was performed as specified by manufacturer instructions. Briefly, cells grown in methionine-free media were pulsed with the AHA amino acid analog for 30 min. Cells were fixed with paraformaldehyde for 15 min, permeabilized with 0.3% triton-PBS for 15 min and then the incorporated analogs were labeled via Click-it chemistry using the Alexa Fluor 488 azide. Cells were then washed with PBS, stained with DAPI and mounted. Each experiment was performed on 3 biological replicates. Significance was assessed with two-tailed Student’s *t*-test.

### qRT-PCR analysis

For semi-quantitative analysis, total RNA was extracted following the manufacturer’s protocols (Qiagen RNeasy mini kit, Qiagen). cDNA was synthetized using a high-capacity RT-kit (Applied Biosystems). Primer sets for these experiments are listed in Supplementary Table 2. Expression levels were determined using SYBR-green mix (Applied Biosystems) and a real-time thermocycler (Applied Biosystems 7500). qRT-PCR values were calculated relative to Gapdh1. Each experiment was performed on 3 biological replicates. Significance was assessed with two-tailed Student’s *t*-test. Statistics: Supplementary Fig. 1c, *P* < 0.02; Fig. 2a, *P* < 0.015; Supplementary Fig. 2d, *P* < 0.01; Fig. 2g, Polr1a, Sirt7, Rrn3, Fbl and Ncl, *P* < 0.01; Fig. 3c, *P* < 0.05; Fig. 3j, *P* < 0.02; Supplementary Fig. 3b, *P* < 0.02.

### siRNA experiments

6 × 10^4^ cells were seeded in a 6-well plate and on the following day treated with 50 nM of ON-TARGET plus Polr1a siRNA- SMARTpool (Dharmacon) and ON-TARGET plus Non-targeting pool (Dharmacon) respectively using DharmaFECT 4 (Dharmacon) transfection reagent overnight. Media was replaced the next day and the cells are treated with TGFβ on the 4th day. The cells were fixed with 4% paraformaldehyde after 48 h and subsequently stained for the respective markers.

### Northern blot analysis

Total RNA from NMuMG cells treated with or without TGFβ was prepared using Tri reagents (Ambion) and loaded on a 1.5% agarose-gel containing 6.5% formaldehyde. Equal amount of RNA was transferred to a nitrocellulose membrane which was probed with radioactively labeled ETS-1 oligonucleotide ETS-5’-agctccccacgggaaagcaatgagtctctc. The oligonucleotide was end-labeled using T4-kinase and P-32 gamma-ATP. Quantifications of Northern blots were conducted using Fuji Phosphoimager. Experiment was performed on 3 biological replicates. Significance was assessed with two-tailed Student’s *t*-test: Supplementary Fig. 2b *P* < 0.028; Supplementary Fig. 2c, *P* < 0.035.

### AgNOR staining

Silver staining of NORs in control cells and TGFβ-treated cells was performed using previously described AgNOR procedures^75^. Briefly, after fixation, incubation with Carnoy’s Solution and rehydration, cells were stained with a freshly prepared AgNOR staining solution for 30 min. After staining, cells were rinsed twice in distilled water, treated with 5% sodium thiosulfate for 2–5 min, rinsed again, and mounted for brightfield microscopy using a Nikon E600 Camera and image capture. Each experiment was performed on 3 biological replicates.

### Chromatin immunoprecipitation

ChIP assays were performed as previously described^28,29^. Formaldehyde cross-linked chromatin obtained from control or TGFβ-treated NMuMG cells were subjected to immunoprecipitation with the autoimmune serum S57299 against Pol I or with antibodies to UBF, SIRT7, Snail1, TIP5, and non-specific mouse IgG as a control (see Supplementary Table 1 for antibody details). DNA-protein complexes were analyzed by qRT-PCR with primers specific for the rDNA promoter, 28 S and 18 S gene in addition to the Snail1 and E-cadherin promoters (primers listed in Supplementary Table 2**)**. The qRT-PCR analysis was performed as previously described. Results are displayed as bars graphs. All ChIP data are presented as a fold induction over IgG control and as relative occupancy. Each experiment was performed on 3 biological replicates. Significance was assessed with two-tailed Student’s t-test. Statistics: Fig. 2f, *P* < 0.03; **h** *P* < 0.006 and *P* < 0.005; **i** *P* < 0.015 and *P* < 0.04; **j** *P* < 0.005; **k** *P* < 0.03; Supplementary Fig. 2l, *P* < 0.0002; Fig. 3d, UBF: control compared to TGFβ, *P* < 0.007; control compared to control + CX-5461, *P* < 0.027; TGFβ compared to TGFβ + CX-5461, *P* < 0.003. Snail1: control compared to TGFβ, *P* < 0.003; control compared to control + CX-5461, NS; TGFβ compared to TGFβ + CX-5461, *P* < 0.02.

### HpaII-methylation assay

NMuMG cells treated with or without TGFβ for 48 h were cross-linked with 1% formaldehyde and chromatin was isolated. The chromatin was sonicated 10 times for 30 s. Cross-linked DNA was purified with phenol/chloroform and precipitated with ethanol. Purified DNA was digested with methylation-sensitive HpaII and MspI separately. DNA was amplified by qRT-PCR using rDNA promoter primers and the ratio between HpaII and Msp I was calculated. Upon methylation, cleavage with HpaII is blocked, while MspI remain unaffected and subsequently induced ratio represent loss of methylation. Each experiment was performed on 3 biological replicates. Significance was assessed with two-tailed Student’s *t*-test. Control compared to TGFβ, *P* < 0.007 (Fig. 2g).

### Invasion assays

The invasive properties of the NMuMG were measured using a Matrigel invasion assay. Cell culture plate inserts (24-well inserts, 0.8-µm pore size; BD Bioscience, Bedford, MA, USA) were coated with Matrigel (1 mg/ml; BD Bioscience). All cells were pre-incubated in media with or without TGFβ for 48 h and 100 nM CX-5461 or 0.01 μg/mL actinomycin D were added at 27 h of TGFβ treatment. Medium with 10% FBS containing 1 × 10^4^ cells were added to the upper chamber insert, and 500 µl of DMEM with 10% FBS was added to the lower chamber. The cells were incubated for 24 h at 37 °C in 5% CO_2_ humidified incubator. Cells that did not pass through the Matrigel were removed from the insert with a cotton swab; invasive cells that crossed the membrane were fixed in 4% paraformaldehyde and subsequently stained with DAPI. The membrane of the insert was cut out and fixed onto a slide with fluorescent mounting medium. The representative number of invasive cells was evaluated by imaging using the Zeiss Confocal microscope and counting invading cells in 10 fields per condition. Each experiment was performed on 3 biological replicates. Significance was assessed with two-tailed Student’s *t*-test. Statistics: Fig. 3g: control compared to TGFβ, *P* < 0.002 TGFβ compared to TGFβ + CX-5461, *P* < 0.001; Supplementary Fig. 3f: control compared to control + ActD, *P* < 0.003; control compared to TGFβ + ActD, *P* < 0.001; Supplementary Fig. 3j, *P* < 0.001.

### Gene expression profiling

NMuMG cells were cultured and treated with TGFβ and CX-5461 as described above. Ribosome profiling was performed as previously described^76^, with the following changes: ribosomes were pelleted through a 1 M sucrose cushion containing 20 mM Mg^2+^, 500 mM NH_4_Cl, 500 mM cycloheximide. Ribosome pellets were resuspended and subunits were dissociated in buffer containing 500 mM KCl, 2 mM puromycin in PBS (pH 7.4), and SUPERase*In™ RNase inhibitor (Thermo; final concentration of 100 U/mL buffer). Ribosomal subunits were pelleted at 438k*g in a TLA 100.3 rotor for 2 h at 4 °C and the supernatant containing ribosome protected fragments was collected and processed for RNA sequencing. Subtractive depletion of rRNA was not performed during library preparation. RNA sequencing was performed on an Illumina HiSeq 2500 instrument at the Genomics Resources Core Facility of Weill Cornell Medicine. Adapter clipping and basic quality filtering were performed on raw sequencing reads using the Fastx toolkit. Reads were then aligned with STAR^77^ to the mouse reference genome GRCm38 from Ensembl^78^. Relative expression levels were calculated using RSEM^6^ and Limma-voom^79^ was applied to determine differential expression (DE) based on the total number of ribosome protected fragments that mapped to annotated mRNAs. We employed a false discovery rate (FDR) threshold of 5%. All RNA sequencing data have been deposited to the Sequence Read Archive under BioProject PRJNA531030. Enrichment of gene ontology categories was determined as previously described^44^.

### Chick developmental experiments

Fertilized chicken eggs were incubated at 37 °C until Hamburger and Hamilton stage 18/19. A small window was made in the eggshell, followed by a hole in the upper membrane through which 200 µL of 200 mM FUrd or 50 mM EdU was injected. Eggs were resealed with tape and incubated at 37 °C for 1 h. Embryos were fixed in 4% paraformaldehyde (PFA) for 1 h at room temperature, washed in PBS then incubated overnight in 30% sucrose and subsequently frozen down in Tissue-Tek O.C.T. (Sakura) for sectioning. Sections were washed in PBS and stained overnight at 4 °C for FUrd and Snail1/2, see (Supplementary Table 1) for antibody details. Secondary antibodies listed previously were applied at 1:1000 dilution for 45 min, followed by DAPI stain. EdU detection was achieved using Click-IT azide-Alexa Fluor 488 (Life Technologies) according to the manufacturer’s protocol. All images were captured with a Zeiss Confocal microscope. Chick experiments were repeated at least 3 times with 2 or more embryos.

### Mice developmental experiments

Pregnant mice were injected intraperitoneally at embryonic day E9.0 with 200 uL PBS containing 2 mg BrdU (Thermofisher, B23151) and 2 mg EU (Thermofisher, E10345). 4 h later mice were sacrificed with isufolran and harvested embryos were fixed for 2 h in 4% PFA at 4 degrees. After overnight incubation in 30% sucrose embryos were embedded in OCT (HistoLab, 45830) and transversally cut in cryosections at 16 µM. Sections were either stored at −20 °C or processed immediately after sectioning. Before primary antibody incubation, sections were treated with DAKO Target Retrieval Solution (Agilent S169984-2) according to the manufacturer’s instructions. Sectioned tissues were incubated with primary Sox10 (Novusbio AF2864) antibody at a dilution of 1:500 overnight at RT in PBS-T (0,1% Tween). For detection of Sox10 488 conjugated Alexa-Fluor secondary antibody produced in donkey (Thermofisher, A-11055 1:1000) was used in combination with secondary BrdU 405 (Novusbio, NBP2- 34784AF405 1:1000) for 3 h at RT diluted in PBS-T. Subsequently, to detect cells that incorporated EU, Click-iT Alexa Fluor 647 Azide (Thermofisher, A10277) was used according to the manufacturer’s instructions. All animal experimentation was performed in accordance with institutional guidelines as detailed in animal protocol #1184203.

### Animal studies and in vivo treatments

The genetically engineered mouse MMTV-PyMT constitutes a faithful model for invasive and metastatic breast carcinoma^59^. Tumors in MMTV- PyMT mice develop through a multistep pathway due to oncogenic activation and end-stage mice present with locally invasive tumors and disseminated disease to lymph nodes and lungs. To investigate the therapeutic utility of inhibition of RNA polymerase I (Pol I) assembly, 8-weeks-old MMTV-PyMT mice were treated once weekly with intraperitoneal injections of CX-5461 at a 50 mg/kg (*n* = 3) and 87 mg/kg (*n* = 3), or vehicle (*n* = 4). Each week before injections tumors were measured. Tumor volume measurements in Fig. 4d, statistically evaluated with ANOVA, *P* < 0.01. At the conclusion of the experiment, following 4 weeks of therapy, mice were sacrificed and primary tumors from all 10 mammary fat pads were harvested. Lungs were also harvested for assessment of metastatic dissemination in Fig. 4f, statistically evaluated with ANOVA, *P* < 0.02. To investigate the inhibitory effect on lung metastasis of E0771 cells by CX-5461, C57BL/6 mice were separated into two groups, one with first treatment 24 h prior and the other 24 h after the tail vein injection of 5 × 10^4^ E0771 cells followed by twice-weekly intraperitoneal injections of CX-5461 at 50 mg/kg in 50 mM sodium phosphate buffer, pH 4.5. Control group was injected with corresponding amount of buffer by mice weight. Mice were sacrificed after two weeks (post-treatment group) or five weeks (pre-treatment group) after the tail vein injection. The number of animals are (for both experiment) *n* = 3 for buffer control, *n* = 4 for CX-5461 treatment. Lungs were harvested for assessment of metastasis by mCherry expression using qRT-PCR relative to β-actin expression. Primers are listed in Supplementary Table 2 (Table S2). Statistics evaluated with two-tailed Students *t*-test (Fig. 4g, *P* < 0.01; *P* < 0.05).

### Mouse and human tissue evaluation

Primary tumors and corresponding lungs from MMTV-PyMT mice at 6-weeks, 8-weeks or 12-weeks old, treated MMTV-PyMT mice (vehicle, 50 mg/kg and 87 mg/kg doses of CX-5461) and E0771 mammary fat pad implanted mice were embedded in paraffin. E0771 cells (1 × 10^6^) were injected in mammary fat pad of C57BL/6 mice and primary tumor and lungs were harvested at week three after injection. Primary tumors and lungs were sectioned at 5 µm, de-paraffinized and stained according to standard protocols. H&E was performed on MMTV-PyMT mice at 6-weeks, 8-weeks or 12-weeks old, treated MMTV-PyMT mice to determine tumor morphology. Early metastatic lesions were identified in lungs of 8-weeks old mice with IHC for PyMT antigen. IHC for identification of expression levels of Pol I (autoimmune serum S57299 against Pol I) and Ki67 (experiments performed in at least 2 or more mice) was performed on 12-week MMTV-PyMT mouse lung metastasis and 3-week E0771 mammary fat pad primary tumor and lung metastasis. In addition, 6-week MMTV-PyMT mice were examined for Rictor, Cytokeratin 8/18 (CK8/18), and ERα expression and in vehicle and CX-5461 treated primary tumor tissues via IHC. Progressive MMTV-PyMT primary tumors at 6-weeks, 8-weeks, and 12-weeks were analyzed for Rictor expression via IHC. H&E and IHC images were taken with a Nikon E600 Camera. Progressive MMTV-PyMT primary tumors at 6-weeks, 8-weeks and 12-weeks were examined for Pol I and Ki67 expression with immunofluorescence. Briefly, slides were de-paraffinized, rehydrated, subjected to antigen retrieval, and incubated with first primary antibody 1 h followed by secondary antibody, then second primary and secondary treatment. Lastly, slides were counterstained with Sudan black and immunofluorescence images were captured with a Zeiss Confocal microscope. Vehicle and CX-5461 treated primary tumor tissues were also analyzed for Snail1/2 and LC3 expression by immunofluorescence. Lung metastases were counted by taking 25 sections from each lung, which were stained with H&E to obtain the number of metastases (Fig. 4f), ANOVA, *P* < 0.02. Mouse tissue sections from primary tumors and corresponding lung metastases from at least 2 mice were IHC-stained from paraffin-embedded tissues from the E0771 medullary adenocarcinomas mouse model^48^ and images of staining expression levels were assayed in the same manner as the MMTV-PyMT model. All MMTV-PyMT animal experimentation was approved by the local ethics committee for animal research (Stockholm Norra, license# N96/11 and Lund, license# M142/13). All E0771 animal experimentation was performed in accordance with institutional, IACUC and AAALAS guidelines, as detailed in our institutional animal protocol #0709-666 A. FFPE (4 µm) sections were obtained from tissue microarrays (TMA) consisting of normal mammary tissue and invasive tumor from 106 patients and stained for Pol I. These studies were followed with whole sections of breast cancer tissues from ER^+^ and TNBC (ER^−^/PR^−^/Her2^−^). Two independent researchers performed blinded scoring of the invasive areas of tissue samples as well as a surgical pathologist (JH), staining intensity was scored on a scale of 0–4 (0 [no staining] – 4 [highest]) for quantification, scoring is represented as an average from all 3 researchers. Figure 5c, Student’s *t*-test, *P* < 0.01. The “Ethics Committee at the Karolinska Institutet”, Stockholm and the “Stockholm Medical Biobank”, approved the study protocol. For all patients whose tumors were included in the immunostaining studies, informed consent forms have been approved and signed. See (Supplementary Table 1) for antibody details.

### Relapse free survival analysis

Relapse Free Survival Analysis was performed using the PROGgene V2 Prognostic Database (http://watson.compbio.iupui.edu/chirayu/proggene/database/?url=proggene)^80^. Each analysis used “breast cancer” as cancer type, “relapse” as survival measure, and bifurcated the gene expression at the median. The data was not divided by or adjusted for any clinical status. The relapse free status was then checked for expression levels of Polr1a and Ubtf, *P* < 0.006.

### Reporting summary

Further information on research design is available in the Nature Research Reporting Summary linked to this article.

## Supplementary information

Supplementary Information

Reporting Summary

## Data Availability

All data will be made available upon request from the authors. The RNA sequencing data presented are available through the Sequence Read Archive BioProject: PRJNA531030.
