# Supplementary material for: Ribosome biogenesis during cell cycle arrest fuels EMT in development and disease
Source: Nat Commun. 2019 May 8;10:2110. doi: 10.1038/s41467-019-10100-8 (PMC6506521; doi:10.1038/s41467-019-10100-8)
Supplement: Supplementary file 1 — Supplementary Information [file 41467_2019_10100_MOESM1_ESM.pdf]

Supplementary Information

Ribosome biogenesis during cell cycle arrest fuels EMT in development and disease

Prakash et al.

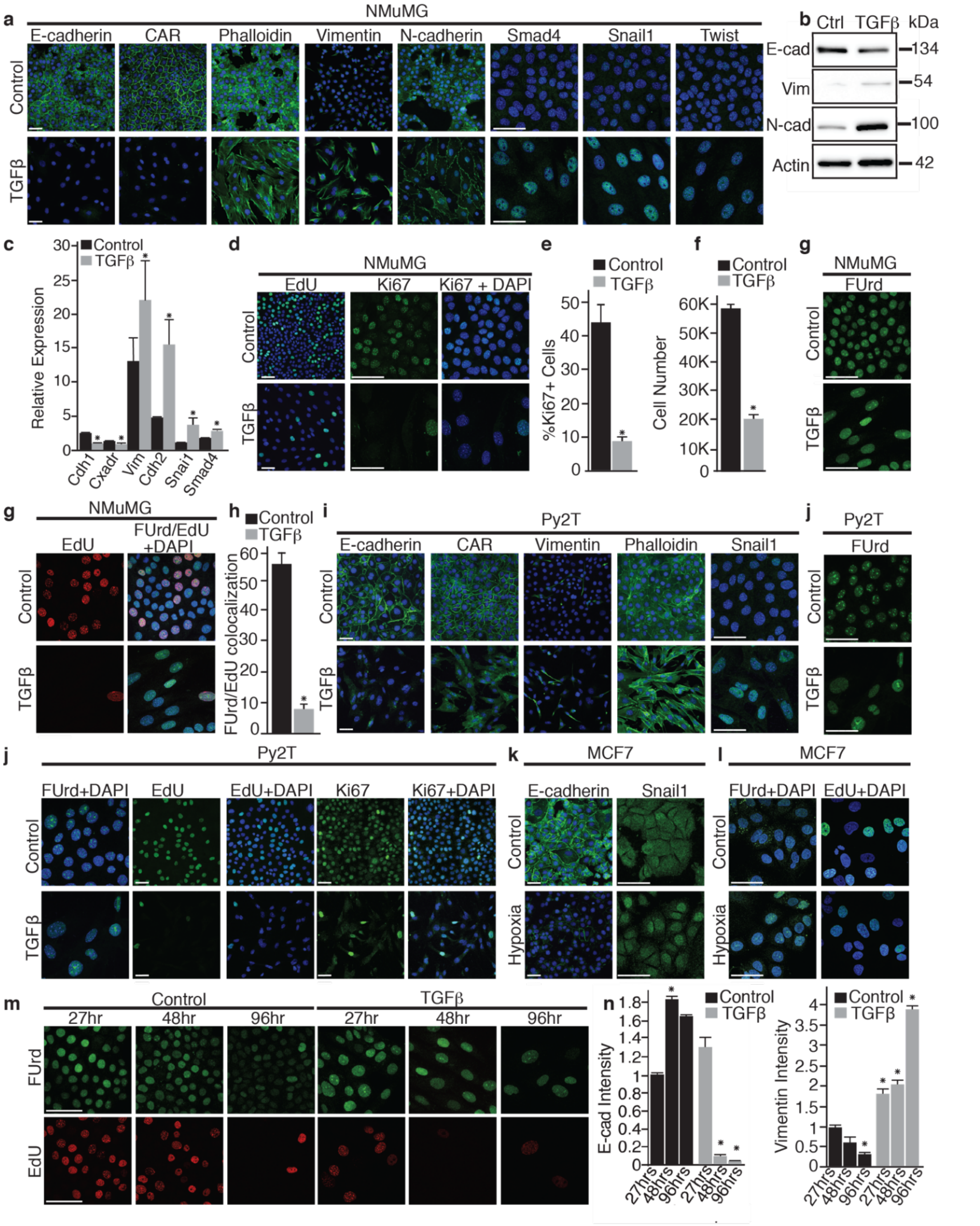

Supplementary Figure 1. Enhanced rRNA synthesis in EMT is independent of cell proliferation. a,

Immunostaining of E-cadherin, CAR, Phalloidin, Vimentin, N-cadherin, Smad4, Snail1, and Twist (green) in proliferating (Control) cells and after 48 hours of TGFβ treatment (TGFβ), merged with DAPI (blue). **b**, E-cadherin (E-cad), Vimentin (Vim) and N-cadherin (N-cad) western blot ± TGFβ in NMuMG cells. Actin serves as a loading control. **c**, qRT-PCR of E-cadherin (Cdh1), CAR (Cxadr), Vimentin (Vim), N-cadherin (Cdh2), Snail1 (Snai1) and Smad4 ± TGFβ in NMuMG cells, $P<0.02$. Error bars ± SD n = 3. **d**, Immunostaining of EdU (green) and Ki67 (green) ± TGFβ, NMuMG cells, DAPI (blue). **e**, Quantification of $Ki67^+$ cells, $P<0.001$. Error bars ± SE, n= 3. **f**, Quantification of NMuMG cell number ± 48 hour TGFβ treatment, $P<0.001$. Error bars ± SD, n= 3 **g**, Co-localization of FUrd, (green) and EdU^+, (red) ± TGFβ, merged with DAPI (blue). **h**, Quantification of FUrd/EdU co-localization in (**g**), $P<0.001$. Error bars ± SE, n=3. **i**, Immunostaining of E-cadherin, CAR, Vimentin, Phalloidin, and Snail1 (all green) in proliferating (Control) and TGFβ-treated (TGFβ) Py2T cells merged with DAPI (blue). **j**, FUrd, EdU and Ki67 (green) in Py2T, alone and merged with DAPI (blue). **k**, Immunostaining of E-cadherin (green) and Snail1 (green) in MCF7 cells under normoxia (Control) or 48-hour hypoxia (Hypoxia) merged with DAPI (blue). **l**, Immunostaining of rRNA synthesis (FUrd, green) and DNA synthesis (EdU, green) in MCF7 cells under normoxia (Control) or 48-hour hypoxia (Hypoxia) merged with DAPI (blue). **m**, Immunostaining of rRNA synthesis (FUrd, green) and DNA synthesis (EdU, red) over 27, 48, and 96 hours in proliferating (Control) and TGFβ-treated (TGFβ) NMuMG cells. **n**, Quantification of E-cadherin (Ecad) and Vimentin time course images from (**m**) in proliferating (Control) and TGFβ– (TGFβ) NMuMG cells. $P<0.05$, $P<0.02$. Asterisk denotes t-test significance. Error bars ± SE, n=3. Scale bars = 50 μm.

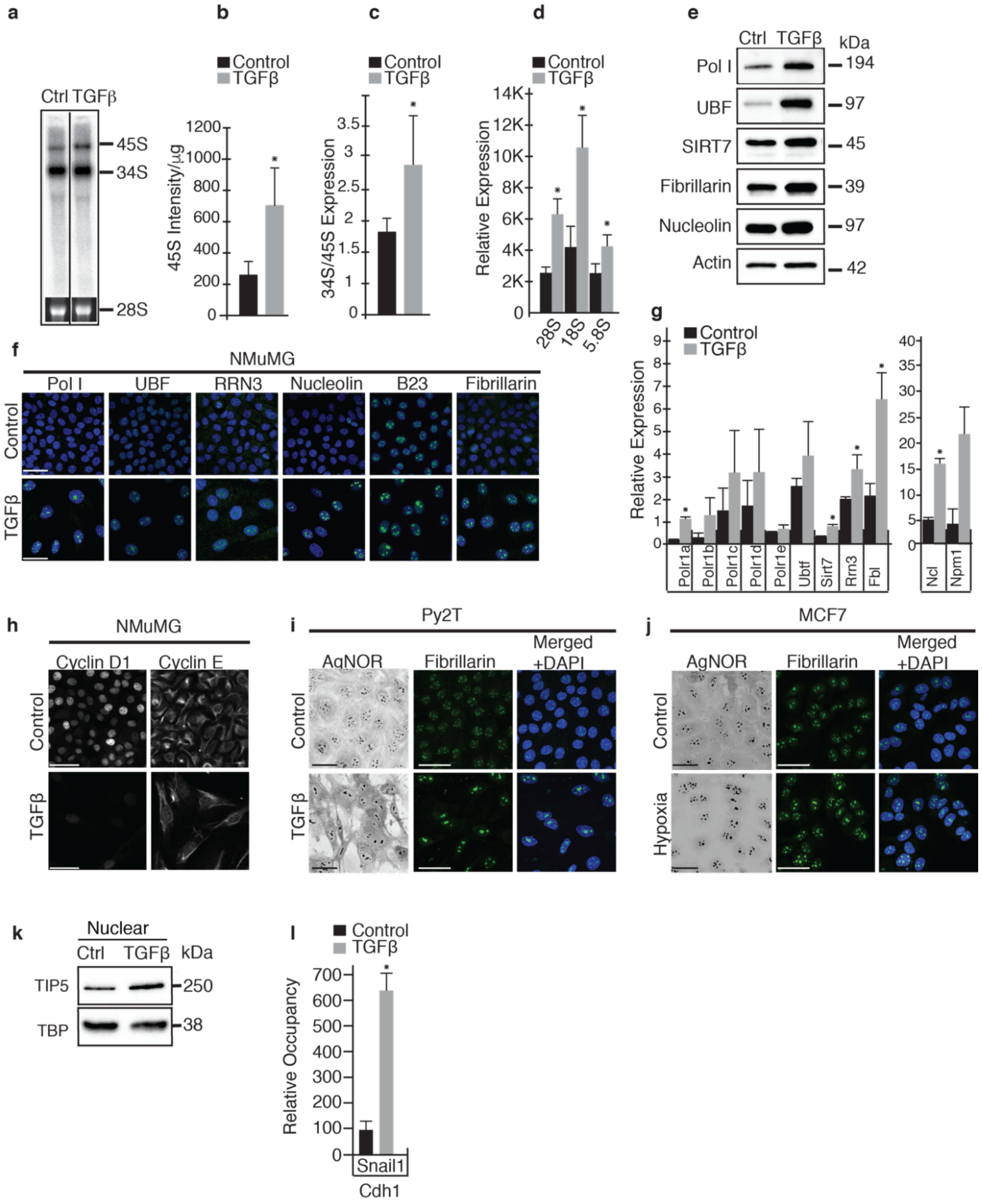

Supplementary Figure 2. Characteristic features of the EMT-associated ribosome biogenesis program. a, Representative Northern blot of NMuMG cells ± TGFβ. **b**, Quantification of Northern blot

analysis of 45S expression in NMuMG cells ± TGFβ. $P<0.03$. **c**, Quantification of Northern blot analysis of 34S/45S expression in NMuMG cells ± TGFβ. $P<0.04$. **d**, qRT-PCR of 28S, 18S and 5.8S rRNA transcripts. $P<0.01$. **e**, Western Blot of Pol I, UBF, SIRT7, Fibrillarin and Nucleolin in NMuMG cells ± TGFβ. Actin serves as a loading control. **f**. Pol I, UBF, RRN3, Nucleolin, B23, and Fibrillarin (all green) immunostaining in NMuMG ± TGFβ, merged with DAPI (blue). **g**, qRT-PCR analysis of Pol I subunits (Polr1a-e), UBF (Ubtf), Sirt7, Rrn3, Fibrillarin (Fbl), Nucleolin (Ncl), and B23 (Npm1) ± TGFβ, NMuMG cells, $P<0.01$ for Polr1a, Sirt7, Rrn3, Fbl, and Ncl. Error bars ± SD, n=3 biological replicates. **h,** Immunostaining of Cyclin D1 and E (white) ± TGFβ, NMuMG cells. **i**, Silver staining of nucleolar organizer regions (NORs) and Fibrillarin expression (green) ± TGFβ, merged with DAPI (blue) in Py2T cells. **j**, Silver staining of nucleolar organizer regions (NORs), immunostaining of Fibrillarin (green) in MCF7 cells ± hypoxia, merged with DAPI (blue). **k,** Western blot of TIP5 nuclear expression levels in NMuMG cells ± TGFβ. TBP serves as a loading control. **l**, Snail1 binding to the E-cadherin (Cdh1) promoter, $P<0.0002$. Error bars ± SE, n= 3. Asterisk denotes t-test significance. Scale bars = 50 μm.

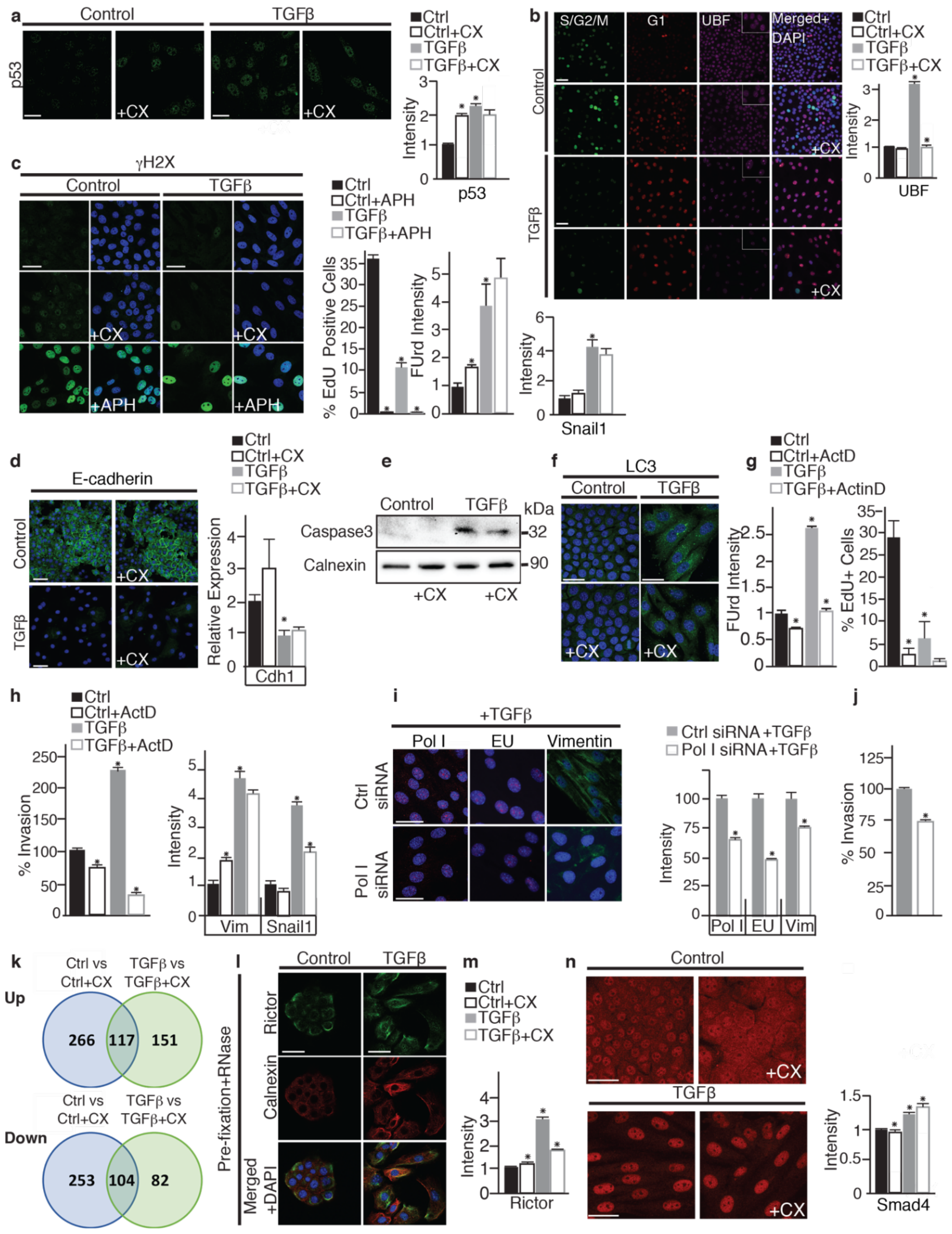

Supplementary Figure 3. Inhibition of Pol I initiation at rDNA operons impairs the EMT program.

All cell culture experiments performed in NMuMG, TGFβ, CX-5461, Actinomycin D (ActD) and

Aphidicolin (APH) labeled in each panel. **a**, p53 expression. Quantification of p53, Control/TGFβ ± CX-5461 treatment, P<0.0001 **b**, Cell cycle analysis using FUCCI system, S/G2/M (geminin, green), G1 (Cdt1, red), UBF (magenta) and DAPI (blue), merged. Quantification of UBF, Control/TGFβ ± CX-5461 treatment, P<0.0001 **c**, γH2X staining (green) with DAPI (blue) ± APH/CX-5461 treatment (blue). Quantification of EdU ± APH treatment, P<0.002. Quantification of FUrd and Snail ± APH treatment, P<0.01. **d**, E-cadherin (green) immunostaining with treatment, merged with DAPI (blue). qRT-PCR of E-cadherin (Cdh1) mRNA expression changes with treatment, P<0.02. **e,** Cleaved Caspase-3 Western Blot. Calnexin serves as a loading control. **f**, LC3 (green) immunostaining merged with DAPI (blue). **g**, Quantification of FUrd and EdU post ActD treatment, P<0.02, P<0.001. **h**, Relative percent invasion from Boyden chamber invasion assay with ActD treatment. P<Control/control+ActD, P<0.003, Control/TGFβ+ActD, P<0.001 Quantification of ActD treated Vimentin and Snail1 (green) immunofluorescence. P<0.03. **i**, Immunostaining of Pol I, EU and Vimentin transfected with Pol I siRNA or Ctrl siRNA in the presence of TGFβ. Quantification of Pol I, EU and Vimentin (Vim) intensity with Pol siRNA or Ctrl siRNA in the presence of TGFβ. P<0.0002. **j**, Relative percent invasion from Boyden chamber invasion assay, with Pol I RNAi or Ctrl siRNA in the presence of TGFβ, P<0.0001. **k**, Venn diagram depicting the overlap of genes upregulated by CX-5461 in proliferating (Control) or TGFβ-treated NMuMG cells. Venn diagram depicting the overlap of genes downregulated by CX-5461 in proliferating (Control) or TGFβ-treated NMuMG cells. **l,** Immunostaining of Rictor (green) and Calnexin (green) ± RNase A (RNase). **m,** Quantification of Rictor in NMuMG cells ± TGFβ± CX-5461 P<0.0001 control compared to (TGFβ and TGFβ compared to (TGFβ+CX-5461). **n,** Immunostaining of Smad4 (green). Quantification of Smad4, Control/TGFβ ± CX-5461 treatment, P<0.0001. For all quantifications: asterisk denotes t-test significance, error bars ± SE, n = 3. Scale bars = 50 μm.

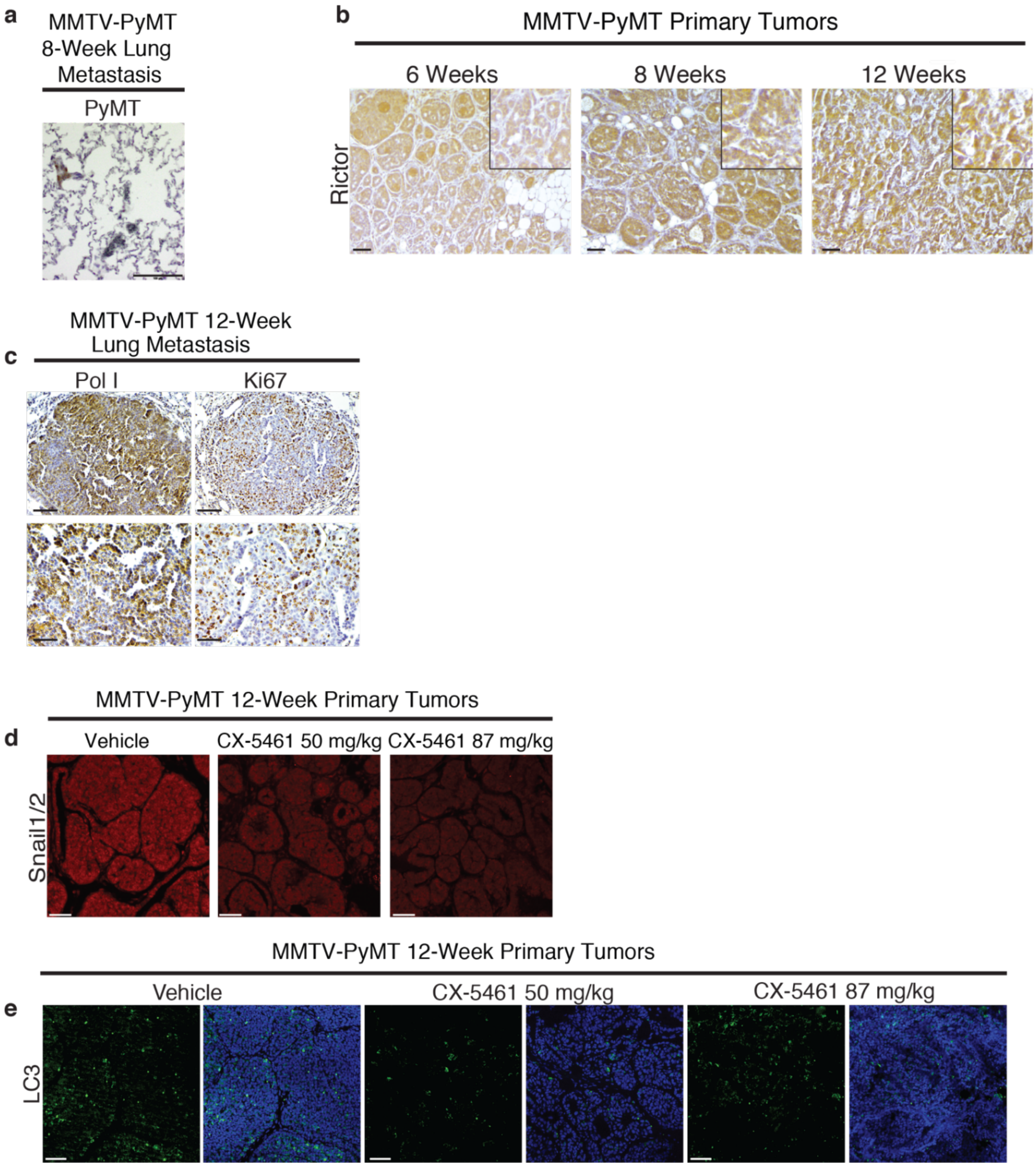

Supplementary Figure 4. Human breast tumors exhibit high-levels of Pol I expression. **a**, IHC staining for PyMT lung micro-metastasis, 8 weeks. **b**, IHC staining of Rictor expression in primary tumor for at 6, 8 and 12 weeks. **c**, IHC staining of 12-week lung metastasis for Pol I and Ki67 expression. **d**, Immunostaining of Snail1/2 (red) in vehicle, 50 mg/kg, or 87 mg/kg CX-5461-treated tumor. **e**, Immunostaining of LC3 (green) in vehicle, 50 mg/kg or 87 mg/kg CX-5461 treated tumors, DAPI (blue).

Supplementary Table 1
Antibody List

Antibody	Order information	Fixation Solution/Time Immunoflourescence	IF/IHC Dilution	Western Blot Dilution	ChIP Dilution
B23	Santa Cruz SC-271737	4% Paraformaldehyde, 15 minutes	1:25		
Beta Actin	Abcam ab1801			1:1000	
BrdU 405	Novusbio NBP2-34784AF405		1:1000		
BrdU	Sigma Aldrich B2531 Clone BU33	4% Paraformaldehyde, 15 minutes	1:1000		
Calnexin	Abcam ab75801			1:1000	
Calnexin	Abcam ab22595	4% Paraformaldehyde, 15 minutes	1:150		
CAR1	gift of Jonas Fuxe Lab	Ethanol, 10 minutes	1:1000		
Cleaved Caspase 3	Cell Signalling 9661			1:1000	
Cytokeratin 8+18	Abcam ab53280		1:100		
E-cadherin	BD Transduction Labs 610182	Ethanol, 10 minutes	1:100	1:300	
Estrogen Receptor alpha	Abcam ab32063		1:250		
Fibrillarin	Abcam ab5821	4% Paraformaldehyde, 15 minutes	1:1000	1:1000	
H3K4Me3	Abcam ab8580				1:40
H3k27AC	Abcam ab4729				1:40
IgG for ChIP	Abcam ab46540				1:400
Ki67	Thermo Scientific RM-9106S1	4% Paraformaldehyde, 15 minutes	1:1000		
LC3	MBL International	4% Paraformaldehyde, 15 minutes	1:1000	1:1000	
N-cadherin	Abcam ab18203	Methanol, 20 seconds	1:1000		
N-cadherin	Abcam ab12221			1:200	
Nucleolin	Abcam ab22758	4% Paraformaldehyde, 15 minutes	1:200	1:300	
Phalloidin	Life technologies A22287	4% Paraformaldehyde, 15 minutes	1:40		
Pol 1_serum	gift of Piergiorgio Percipalle Lab			1:300	1:500
Polr1e	Atlas Antibodies HPA022527		IHC 1:500		
p53	Santa Cruz SC-126	4% Paraformaldehyde, 15 minutes	1:50		
RICTOR antibody [7B3]	Abcam ab104838	4% Paraformaldehyde, 15 minutes	1:150		
RPA194	Abcam ab48385	4% Paraformaldehyde, 15 minutes	1:25		
RRN3	Abcam ab169511	4% Paraformaldehyde, 15 minutes	1:25		
Sirt7	Abcam ab62748			1:200	1:40
Smad 4	Santa Cruz B-8 SC-81417	4% Paraformaldehyde, 15 minutes	1:25	1:50	
Snail 1	gift from Garcia de Herrero lab	4% Paraformaldehyde, 15 minutes	undilluted		
Snail1/2	Abcam ab85936	4% Paraformaldehyde, chick 1hr	1:100		
SNAI 1 (H-130)	Santa Cruz SC-28199				1:27
Sox10	Novusbio AF2864		1:500		
TBP	Abcam ab51841			1:1000	
TIP5	Life technologies 49-1037			1:1000	1:25
TIP5	Diagenode C15310090			1:1000	
UBF	Santa Cruz F-9 13125	4% Paraformaldehyde, 15 minutes	1:100	1:300	
UBF	Santa Cruz H-300, 9131				1:40
Vimentin	Abcam ab45939	Methanol, 20 seconds	1:1000	1:200	

Supplementary Table 2
Primer List

**RT-PCR**			
**protein**	**gene**	**Forward**	**Reverse**
**Car**	Cxadr	ccctggggttgcaaataag	gatccatccacgaagcatct
**E-cadherin**	Cdh1	atcctcgccctgctgatt	accaccgttctcctccgta
**Fibrillarin**	Fbl	aggaggatgcccttgtcac	ggttccaggctctgtactcaa
**Gapdh**	Gapdh	gtatgactccactcacggcaaa	ggtctcgctcctggaagatg
**N-cadherin**	Cdh2	gccatcatcgctatccttct	ccgtttcatccataccacaaa
**Nucleolin**	Ncl	catggtgaagctcgcaaag	tcactatcctcttccacctcctt
**Nucleophosmin**	Npm1	gaaaaaggcggttctcttcc	tttcctccactgccagagat
**Vimentin**	Vim	tgcgccagcagtatgaaa	gcctcagagaggtcagcaaa
**PolI A**	Polr1a	cgttcatcgaggactaccagt	tcttcatcagtgggagcttca
**PolI B**	Polr1b	atggcgacccttactacagc	gttgtccacaacacagttttcttta
**PolIC**	Polr1c	cggtaactacgccggttatg	atttgcaccacgtccacac
**PolID**	Polr1d	agcgccacctgaggattt	ttgaggtcttcaatcctgatatttt
**PolIE**	Polr1e	gaaaggtcacatcggaagaca	gggcagagacttcagcattt
**Rrn3**	rrn3	gcctcctgccatgtacagtt	caaaaatgcttctgcaaatcc
**Sirt7**	Sirt7	tgtcatgcaactcctcatgaat	ggtcgccaaggagaagatt
**Smad4**	Smad4	gagaacattggatggacgact	cacagacgggcatagatcac
**Snail**	Snai1	cttgtgtctgcacgacctgt	caggagaatggcttctcacc
**Ubf**	Ubtf	tcactagcacccacttgctc	gcttctccgttcattctcca
**Rictor**	Rictor	tgcgatattggccatagtga	acccggctgctcttacttct
	**rRNA**	**Forward**	**Reverse**
	**45S rRNA (pre-rRNA)**	gcttgtttctcccgattgc	cgcgaacaactgagaaaagt
	**28S rRNA**	agtcgggttgcttgggaatgc	cccttacggtacttgttgact
	**18S rRNA**	cggctaccacatccaagg	tacagggcctcgaaagagtc
	**5.8S rRNA**	actcttagcggtggatcactc	aagcgacgctcagacagg
**Mouse Metastasis Studies**		**Forward**	**Reverse**
	**mCherry**	ctcccacaacgacgactaca	cttgtacagctcgtccatgc
	**Beta-Actin**	tcctggcctcactgtccac	gcaagtgcttctaggcggac
**ChIP**		**Forward**	**Reverse**
	**rDNA promoter**	gacctgtcggtcttatcagttc	ccggacctcaaaggaacaac
	**18S**	cggctaccacatccaagg	tacagggcctcgaaagagtc
	**28S**	agtcgggttgcttgggaatgc	cccttacggtacttgttgact
	**E-cadherin promoter**	agacaggggtggaggaagtt	accagtgagcagcgcagag
