# Supplementary material for: Ribosome biogenesis during cell cycle arrest fuels EMT in development and disease
Source: Nat Commun. 2019 May 8;10:2110. doi: 10.1038/s41467-019-10100-8 (PMC6506521; doi:10.1038/s41467-019-10100-8)
Supplement: Supplementary file 2 — Reporting Summary [file 41467_2019_10100_MOESM2_ESM.pdf]

natureresearch

Corresponding author(s): C. Theresa Vincent and Scott C. Blanchard

Last updated by author(s): Apr 5, 2019

Reporting Summary

Nature Research wishes to improve the reproducibility of the work that we publish. This form provides structure for consistency and transparency in reporting. For further information on Nature Research policies, see Authors & Referees and the Editorial Policy Checklist.

Statistics

For all statistical analyses, confirm that the following items are present in the figure legend, table legend, main text, or Methods section.

n/a	Confirmed	
☐	☒	The exact sample size (n) for each experimental group/condition, given as a discrete number and unit of measurement
☐	☒	A statement on whether measurements were taken from distinct samples or whether the same sample was measured repeatedly
☐	☒	The statistical test(s) used AND whether they are one- or two-sided<br>*Only common tests should be described solely by name; describe more complex techniques in the Methods section.*
☒	☐	A description of all covariates tested
☒	☐	A description of any assumptions or corrections, such as tests of normality and adjustment for multiple comparisons
☐	☒	A full description of the statistical parameters including central tendency (e.g. means) or other basic estimates (e.g. regression coefficient) AND variation (e.g. standard deviation) or associated estimates of uncertainty (e.g. confidence intervals)
☐	☒	For null hypothesis testing, the test statistic (e.g. F, t, r) with confidence intervals, effect sizes, degrees of freedom and P value noted<br>*Give P values as exact values whenever suitable.*
☒	☐	For Bayesian analysis, information on the choice of priors and Markov chain Monte Carlo settings
☒	☐	For hierarchical and complex designs, identification of the appropriate level for tests and full reporting of outcomes
☒	☐	Estimates of effect sizes (e.g. Cohen's d, Pearson's r), indicating how they were calculated

Our web collection on statistics for biologists contains articles on many of the points above.

Software and code

Policy information about availability of computer code

Data collection	N/A
Data analysis	N/A

For manuscripts utilizing custom algorithms or software that are central to the research but not yet described in published literature, software must be made available to editors/reviewers. We strongly encourage code deposition in a community repository (e.g. GitHub). See the Nature Research guidelines for submitting code & software for further information.

Data

Policy information about availability of data

All manuscripts must include a data availability statement. This statement should provide the following information, where applicable:

- Accession codes, unique identifiers, or web links for publicly available datasets
- A list of figures that have associated raw data
- A description of any restrictions on data availability

All data will be made available upon request from the authors. The RNA sequencing data presented are available through the Sequence Read Archive BioProject: PRJNA531030.

Field-specific reporting

Please select the one below that is the best fit for your research. If you are not sure, read the appropriate sections before making your selection.

☒ Life sciences ☐ Behavioural & social sciences ☐ Ecological, evolutionary & environmental sciences

October 2018

Life sciences study design

All studies must disclose on these points even when the disclosure is negative.

Sample size	Sample size was chosen by an estimate of the number of animals needed to detect a 30% difference in the primary endpoints (tumor growth rate and metastatic colonization), based on the experience of the authors.
Data exclusions	No data was excluded from the analysis
Replication	Each experiment was performed in three biological replicates.
Randomization	No randomization was used
Blinding	The investigator performing animal experiments was aware of which therapy was administered to each group.

Reporting for specific materials, systems and methods

We require information from authors about some types of materials, experimental systems and methods used in many studies. Here, indicate whether each material, system or method listed is relevant to your study. If you are not sure if a list item applies to your research, read the appropriate section before selecting a response.

Materials & experimental systems

n/a	Involved in the study	
☐	☒	Antibodies
☐	☒	Eukaryotic cell lines
☒	☐	Palaeontology
☐	☒	Animals and other organisms
☐	☒	Human research participants
☒	☐	Clinical data

Methods

n/a	Involved in the study	
☒	☐	ChIP-seq
☒	☐	Flow cytometry
☒	☐	MRI-based neuroimaging

Antibodies

Antibodies used	See Table 1.
Validation	All antibodies used in this study are commercially available and validated.

Eukaryotic cell lines

Policy information about cell lines

Cell line source(s)	Methods; ATCC, RIKEN BRC and collaborators
Authentication	All cell lines were purchased from ATCC, RIKEN BRC or verified by collaborators
Mycoplasma contamination	Cells were routinely tested for mycoplasma
Commonly misidentified lines (See ICLAC register)	N/A

Animals and other organisms

Policy information about studies involving animals; ARRIVE guidelines recommended for reporting animal research

Laboratory animals	Methods. MMTV-PyMT (FVB/N), C57 BL/6
Wild animals	N/A
Field-collected samples	N/A
Ethics oversight	Methods; (Stockholm Norra, license# N96/11 and Lund, license# M142/13), #0709-666A, #1184203

Note that full information on the approval of the study protocol must also be provided in the manuscript.

Human research participants

Policy information about studies involving human research participants

Population characteristics	N/A
Recruitment	N/A
Ethics oversight	Methods; The “Ethics Committee at the Karolinska Institutet", Stockholm and the "Stockholm Medical Biobank"

Note that full information on the approval of the study protocol must also be provided in the manuscript.
